# Oxidation Resistance 1 Modulates Glycolytic Pathways in the Cerebellum via an Interaction with Glucose-6-Phosphate Isomerase

**DOI:** 10.1007/s12035-018-1174-x

**Published:** 2018-06-15

**Authors:** Mattéa J. Finelli, Teresa Paramo, Elisabete Pires, Brent J. Ryan, Richard Wade-Martins, Philip C. Biggin, James McCullagh, Peter L. Oliver

**Affiliations:** 1 Department of Physiology, Anatomy and Genetics, University of Oxford, Parks Road, Oxford, OX1 3PT UK; 2 Department of Biochemistry, University of Oxford, Parks Road, Oxford, OX1 3QU UK; 3 Chemistry Research Laboratory, Department of Chemistry, University of Oxford, Mansfield Road, Oxford, OX1 3TA UK; 4 Oxford Parkinson’s Disease Centre, University of Oxford, South Parks Road, Oxford, OX1 3QX UK; 5 MRC Harwell Institute, Harwell Campus, South Parks Road, Oxford, Oxfordshire OX11 0RD UK

**Keywords:** Oxidative stress, Glucose metabolism, Neurodegeneration, Cerebellum, Mouse

## Abstract

Glucose metabolism is essential for the brain: it not only provides the required energy for cellular function and communication but also participates in balancing the levels of oxidative stress in neurons. Defects in glucose metabolism have been described in neurodegenerative disease; however, it remains unclear how this fundamental process contributes to neuronal cell death in these disorders. Here, we investigated the molecular mechanisms driving the selective neurodegeneration in an ataxic mouse model lacking oxidation resistance 1 (Oxr1) and discovered an unexpected function for this protein as a regulator of the glycolytic enzyme, glucose-6-phosphate isomerase (GPI/Gpi1). Initially, we present a dysregulation of metabolites of glucose metabolism at the pre-symptomatic stage in the *Oxr1* knockout cerebellum. We then demonstrate that Oxr1 and Gpi1 physically and functionally interact and that the level of Gpi1 oligomerisation is disrupted when Oxr1 is deleted in vivo. Furthermore, we show that Oxr1 modulates the additional and less well-understood roles of Gpi1 as a cytokine and neuroprotective factor. Overall, our data identify a new molecular function for Oxr1, establishing this protein as important player in neuronal survival, regulating both oxidative stress and glucose metabolism in the brain.

**Electronic supplementary material:**

The online version of this article (10.1007/s12035-018-1174-x) contains supplementary material, which is available to authorized users.

## Introduction

The human brain consumes 20% of the total energy utilised by the body at rest while accounting for only 2% of the total mass [1]. To meet this particularly high energy demand, the brain uses glucose as the predominant energy substrate under physiological conditions [2]. Glucose is processed by oxidative catabolism (glycolysis and the pentose phosphate pathway (PPP)) in addition to oxidative phosphorylation (tricarboxylic acid (TCA) cycle) in mitochondria. In contrast to other cell types, some subtypes of neurons preferentially metabolise glucose via the PPP to reduce the level of potentially damaging reactive oxygen species (ROS) that are produced by oxidative phosphorylation [2].

It has long been recognised that the brain is particularly vulnerable to changes in glucose metabolism, and this apparent sensitivity may be an important feature in neurological disease. For example, imaging studies of patients at the early symptomatic stages of amyotrophic lateral sclerosis (ALS) demonstrate hypermetabolism (an increase in glucose use) in the central nervous system (CNS), which correlates with disease progression [3, 4]. Conversely, hypometabolism is observed in the striatum of patients affected by Huntington’s disease (HD) [5] or in specific brain areas affected in patients with Alzheimer’s disease (AD) [6]. Furthermore, in addition to glucose usage, the expression of metabolic enzymes has been shown to be disrupted in neurodegenerative conditions; reduced expression of α-glucosidase—the enzyme that degrades glycogen to glucose—is observed in laser-captured motor neurons from post-mortem ALS spinal cords [7], and glucose-6-phosphate isomerase (Gpi1) (also termed GPI in human, phosphoglucose isomerase (PGI), neuroleukin (NLK), or autocrine motility factor (AMF)) is upregulated in the brain of a mouse model of HD [8]. Similarly, the activity of glycolytic enzymes is often deregulated in neurodegenerative diseases; for instance, the activities of pyruvate kinase and lactate dehydrogenase are increased in brain areas affected in AD, while the activities of phosphofructokinase and phosphoglycerate mutase are decreased in the same anatomical regions [9, 10]. Taken together, these studies suggest that the deregulation of glucose metabolism may be an important feature of the neurodegenerative disease process.

Oxidative stress is also a well-established contributing factor in neurodegeneration, with evidence that damage caused by excess ROS is a key aspect of the region-specific pathology observed in disease (reviewed in [11]). Crucially, glucose metabolism and oxidative stress are two pathways that are functionally intertwined; ROS inhibits glycolytic enzymes, leading to activation of the oxidative arm of the PPP to generate NADPH that provides the reducing power of the antioxidant system (reviewed in [11]). Thus, proteins that function at the intersection of these two critical pathways may provide a route to modulating neuronal metabolism as a novel therapeutic strategy in neurodegeneration.

The oxidation resistance 1 (Oxr1) gene was first identified in a human cDNA library screen for genes that could rescue the DNA oxidation repair-defective phenotype of a spontaneous *Escherichia coli* mutant [12]. It has since been defined as an important oxidative stress-associated neuroprotective factor (reviewed in [13]). We have described previously that mice lacking *Oxr1* display degeneration of cerebellar granule cells (CGCs) correlating with a progressive and rapid deterioration of motor coordination and a shortened lifespan [14]. In addition, it has been established that the expression level of Oxr1 confers sensitivity to oxidative stress-associated insults; neurons over-expressing Oxr1 are protected, while Oxr1 knockdown increases the vulnerability of neurons to degeneration [14]. Significantly, enhanced Oxr1 levels are protective against neuronal death in both cellular and mouse models of ALS [15–17], and these protective properties appear to rely on a fully-functional TLDc domain, a highly evolutionary conserved C-terminal motif in OXR1 [13, 16, 18]. This particular domain is found in a family of proteins that includes nuclear receptor coactivator 7 (NCOA7) [16, 18] and TBC1 domain family member 24 (TBC1D24) [16, 19]. In particular, TBC1D24 is mutated in a range of neurological disorders characterised by seizures, neurodegeneration, and hearing loss [20, 21]. Yet, despite the obvious significance of the TLDc family in the CNS, the molecular function of these proteins remains unclear.

Here, we investigated the molecular mechanisms of Oxr1 function in the CNS, and the pre-symptomatic pathways that drive the selective cerebellar neurodegeneration observed in mice lacking *Oxr1*. Using a combination of metabolomic and biochemical approaches, we demonstrate an unexpected role for Oxr1 as a regulator of the glycolytic enzyme, Gpi1. We also show that Oxr1 is a direct protein interactor of Gpi1. Furthermore, Oxr1 appears to modulate key functional aspects of Gpi1, including the formation of multimeric species and the role of the protein as a cytokine. Together, these data suggest that the disruption of normal Gpi1 function and of the glycolytic response in the cerebellum contributes to the selective neurodegeneration in *Oxr1* knockout mice.

## Results

### Loss of Oxr1 Disrupts Normal Glycolytic Metabolism in the Cerebellum

**Fig. 1:**
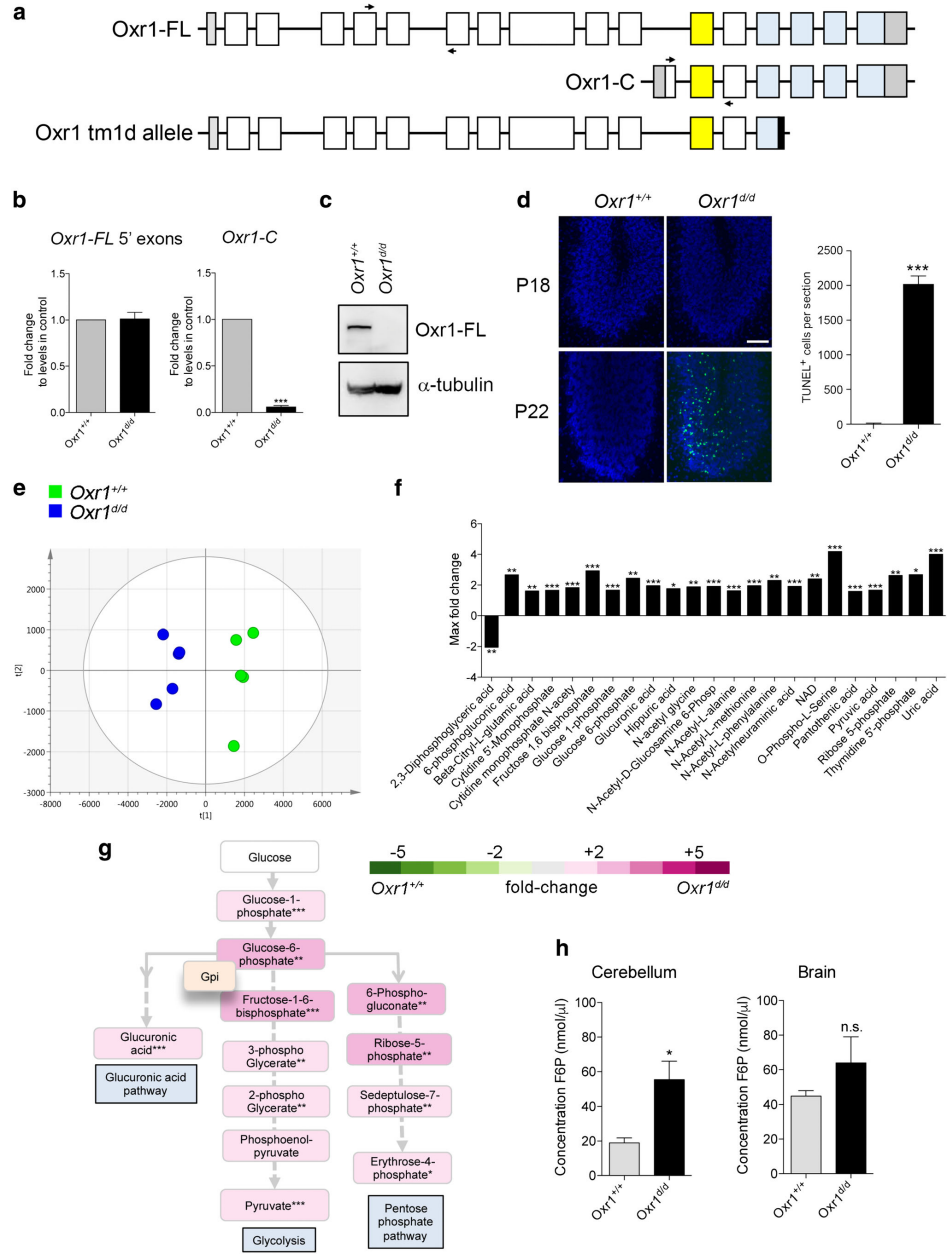
Glucose metabolism is deregulated in Oxr1 knockout mice. **a** Schematic indicating the exon structure of the longest full-length (FL) and shortest C-terminal (C) Oxr1 isoforms with the relative position of qRT-PCR primers indicated (arrows). The Oxr1 tm1d knockout allele is also shown that truncates all Oxr1 protein isoforms. Coding exons (white), UTRs (grey), an alternatively-spliced exon (yellow), and the TLDc domain (blue) are shown. Not to scale. **b** qRT-PCR of 5′ exons of *Oxr1-FL* and the *Oxr1-C* isoform in the cerebellum of *Oxr1*^*d/d*^ mice (*N* = 4 animals per group). **c** Western blot of Oxr1-FL protein in cerebellum tissue of homozygous *Oxr1*^*d/d*^ mice compared to a wild-type control (*Oxr1*^*+/+*^); alpha-tubulin was used as a loading control. **d** Representative TUNEL staining of cerebellum sections from *Oxr1*^*d/d*^ and littermate control mice at P18 compared to disease end-stage at P24 with quantification of TUNEL-positive cells at P24 (*N* = 3 animals per group). Scale bar: 200 μm. **e** PCA analysis of metabolite profiling; each point on the PCA plot represents an individual sample. PCA analysis of metabolite profiles from cerebella of P18 *Oxr1*^*d/d*^ (blue) compared to *Oxr1*^*+/+*^mice (green) (*N* = 5 animals per group). **f** Twenty-three metabolites significantly dysregulated by more than 1.6-fold in the cerebella of P18 *Oxr1*^*d/d*^ compared to *Oxr1*^*+/+*^ mice. Fold-changes are based on per-run abundances for a specific metabolite which were then grouped by experimental condition (*N* = 4-5 animals per group). **g** Metabolites dysregulated in the *Oxr1*^*d/d*^ cerebellum that are featured in pathways downstream of glucose-6-phosphate isomerase. The fold-changes between *Oxr1*^*+/+*^ and *Oxr1*^*d/d*^ mice of the indicated metabolites are colour-coded. The position of the glycolytic enzyme glucose-6-phosphate isomerase (Gpi1) in the pathway is shown. A more detailed representation of the data is shown in Supplementary Fig. 2. **h** Fructose-6-phosphate levels are increased in the cerebellum but not the remaining brain tissue of *Oxr1*^*d/d*^ mice compared to littermate *Oxr1*^*+/+*^ controls (*N* = 4 animals per group). Panels **b**, **d**, and **h**: *t* test, panel **f**, one-way ANOVA: *p < 0.05, **p < 0.01, ***p < 0.001

We have described previously an *Oxr1* mouse model in which the TLDc domain—present in all Oxr1 isoforms—is disrupted by insertion of a lacZ reporter [16]. Based on the same mouse line, we generated mice with constitutive deletion of two exons of the TLDc domain leading to truncation of the protein (Fig. 1a and Supplementary Fig. 1). In mice homozygous for this mutant *Oxr1*^*tm1d*^ allele (henceforth *Oxr1*^*d/d*^), the shortest isoform (*Oxr1-C*) was not expressed in the brain, while the N-terminal end of the full length isoform (*Oxr1-FL*) was still transcribed (Fig. 1b). Importantly, however, the Oxr1-FL isoform was not detectable at the protein level in *Oxr1*^*d/d*^ mutants compared to wild-type littermate controls (*Oxr1*^*+/+*^) (Fig. 1c). *Oxr1*^*d/d*^ mice appear initially to be phenotypically normal until post-natal day (P)19 when they begin to display an ataxic phenotype and selective CGC degeneration (Fig. 1d); this is an identical time-course of cerebellar-specific neurodegeneration as the two previously described mouse models lacking *Oxr1*: the lacZ insertional mutant [16] and the original mutant (*bella*) we described in which the entire *Oxr1* locus was deleted [14].

Regulation of energy metabolism in the cerebellum is critical for granule cells, which have been predicted to consume most of the glucose in this brain region to generate resting and action potentials and maintain post-synaptic receptors [22]. Thus, given that granule cells are specifically affected in *Oxr1*^*d/d*^ mice, we hypothesised that dysregulation of glucose metabolism may drive the neurodegeneration and ataxia observed. To investigate this, metabolic profiling was performed using anion-exchange tandem mass spectrometry from cerebella of pre-symptomatic P18 *Oxr1*^*d/d*^ and wild-type littermate controls [23–25].

Authentic standards were used to identify metabolites associated with central metabolism including glycolysis, PPP, and the TCA cycle. We used principal component analysis (PCA) to model identified and unidentified compounds and their abundances. Compound abundances across all samples in the dataset were plotted in multi-dimensional space as represented on the PCA plot, where the first two principle components of the PCA represent the projections that best discriminate the samples (Fig. 1e). It can be seen that this discrimination divides *Oxr1*^*+/+*^ and *Oxr1*^*d/d*^ extracts, suggesting that there are significant differences in metabolite composition between these two groups. No sample outliers were shown in this dataset, and the principle components 1 and 2 account for 34 and 15% of the total variance in the dataset, respectively. Therefore, these analyses suggested that, in the cerebellum, the two genotypes possessed distinct pre-symptomatic metabolite profiles. Using this approach in combination with calculated fold-changes in abundance, we identified 111 metabolites; of the 52 metabolites significantly deregulated (*p* value < 0.05), 45 were elevated in the *Oxr1*^*d/d*^ samples compared to wild-type, while 7 were depleted (Fig. 1f and Supplementary Table 1). Interestingly, glycolytic metabolites showed generally elevated levels in the *Oxr1*^*d/d*^ cerebellum samples compared to *Oxr1*^*+/+*^; these included those in the first catalytic steps of glycolysis as well as pyruvate, the last metabolite in the process (Fig. 1g and Supplementary Fig. 2).

Given that the initial steps of glycolysis—and in particular glucose-6-phosphate and fructose-1,6-bisphosphate—were significantly enriched, we decided to help validate these observations using an alternative biochemical assay on an independent set of tissues. Thus, we chose to quantify the levels of fructose-6-phosphate, the first metabolic product in glycolysis formed from glucose-6-phosphate, as the metabolomics analysis was not able to quantify this metabolite reliably. Consistent with the deregulation of glycolysis observed by metabolic profiling, we showed a significant increase of 2.9-fold in the cerebellum of *Oxr1*^*d/d*^ mice compared to their littermate controls at P18 (Fig. 1h). Interestingly, a non-significant increase was detectable in the remaining brain tissue of the same animals (Fig. 1h), suggesting a specific and more pronounced dysregulation of this metabolite in the cerebellum at a pre-symptomatic stage of *Oxr1*^*d/d*^ mice, in-line with the selective neurodegeneration observed. In summary, both metabolic profiling by mass spectrometry and a biochemical assay from the *Oxr1*^*d/d*^ cerebellum showed a clear imbalance in the levels of energetic metabolites prior to symptom onset, especially those in the glycolytic pathway and PPP (Supplementary Fig. 2).

### Oxr1 Interacts with Glucose-6-Phosphate Isomerase

**Fig. 2:**
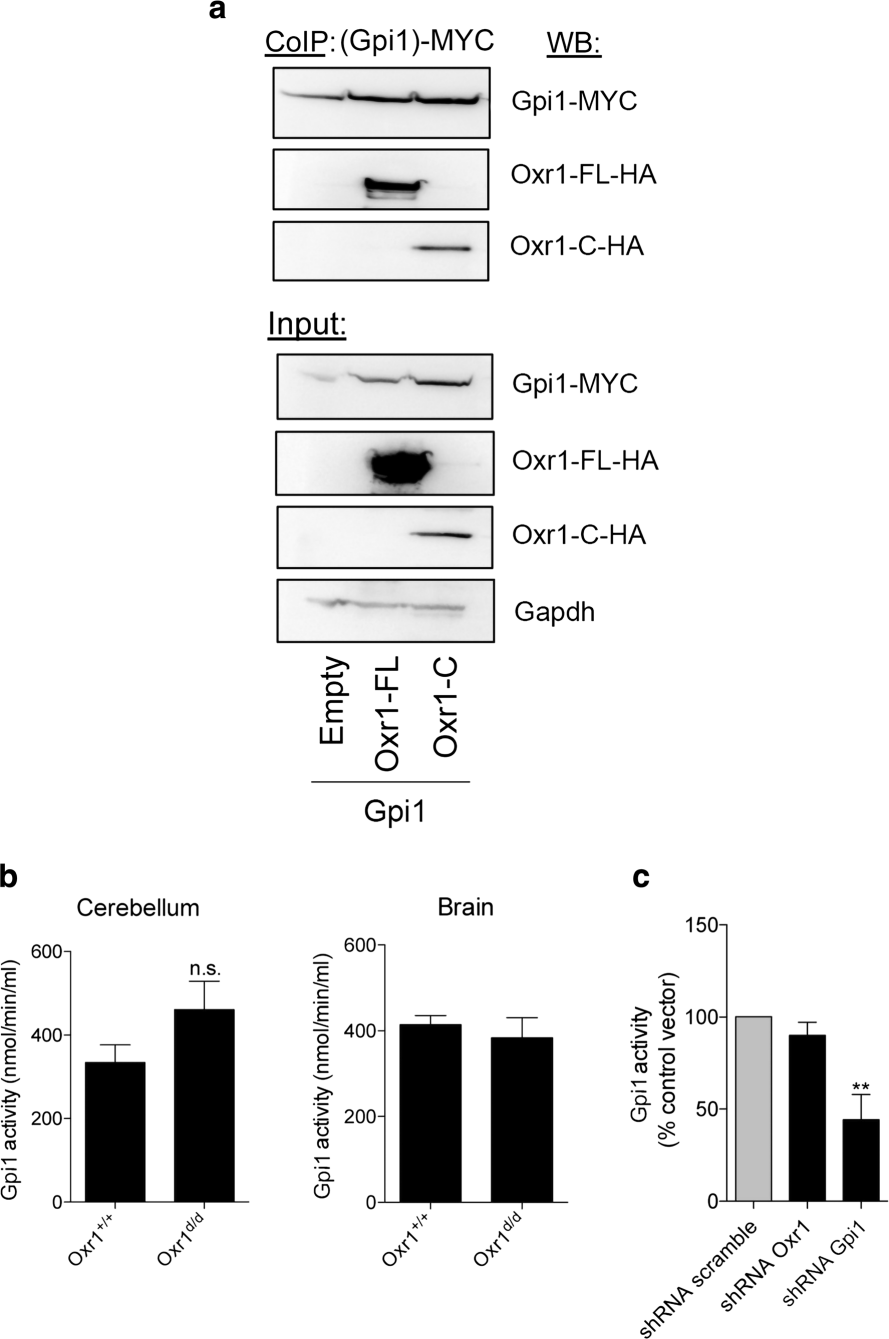
Oxr1 interacts with the glycolytic enzyme, glucose-6-phosphate isomerase. **a** Co-immunoprecipitation in HeLa cells over-expressing MYC-tagged Gpi1 with HA-tagged Oxr1-FL or Oxr1-C. **b** Gpi1 activity from whole cerebellum or remaining brain tissue of *Oxr1*^*d/d*^ mice compared to *Oxr1*^*+/+*^ controls (*N* = 4–8 animals per group). **c** Gpi1 activity in N2a cells transfected with an shRNA against either *Oxr1* or *Gpi1* or corresponding control vector (shRNA scramble) (*N* = 4 independent repeats). Panel **b**: *t* test, panel **c**: one-way ANOVA; **p < 0.01

We reasoned that because multiple parallel pathways associated with glucose-6-phosphate were affected in the *Oxr1*^*d/d*^ cerebellum (Fig. 1g), Oxr1 might act on a protein that metabolises glucose-6-phosphate, such as glucose-6-phosphate isomerase (GPI in human/Gpi1 in mouse). GPI is not only a key enzyme at the crossroads of the glycolytic, PPP, and glucuronic acid pathways [26], it can also act as a chemokine that stimulates cell proliferation and migration [27–29]. Moreover, mutations in GPI have been identified in patients with neuromuscular dysfunction and mental retardation [30–34]. Thus, given the potential role played by GPI in the CNS, we first tested whether Oxr1 and Gpi1 proteins could physically interact. In a cell line co-expressing either the full-length (Oxr1-FL) or the shortest TLDc domain-containing (Oxr1-C) isoforms with Gpi1, a direct interaction could be observed with both isoforms by co-immunoprecipitation (Fig. 2a). These data suggest not only that both Oxr1 isoforms bind to Gpi1 but also that the TLDc domain is critical for this interaction. Unfortunately, we were unable to reproducibly immunoprecipitate Gpi1 with Oxr1 in vivo due to a lack of reliable antibodies. Therefore, we investigated next whether loss of Oxr1 in vivo would affect the enzymatic activity of Gpi1. In cerebellar tissue, Gpi1 activity was increased but not significantly altered in *Oxr1*^*d/d*^ mice compared to control littermates, and no change was detected in the remainder of the brain from the same animals (Fig. 2b). To focus on the role of Oxr1 and Gpi1 in neurons without the potential confound of other cell types, we next tested whether Oxr1 levels would affect Gpi1 activity in a neuronal cell line, Neuro2a (N2a). To mimic the *Oxr1* knockout condition, we transfected N2a cells with a shRNA construct against *Oxr1* to significantly reduce *Oxr1* levels (Supplementary Fig. 3). Similar to what we observed in vivo, reduction of *Oxr1* expression levels did not significantly affect Gpi1 activity (Fig. 2c).

### Loss of Oxr1 Influences Glycolytic Function Under Oxidative Stress

**Fig. 3:**
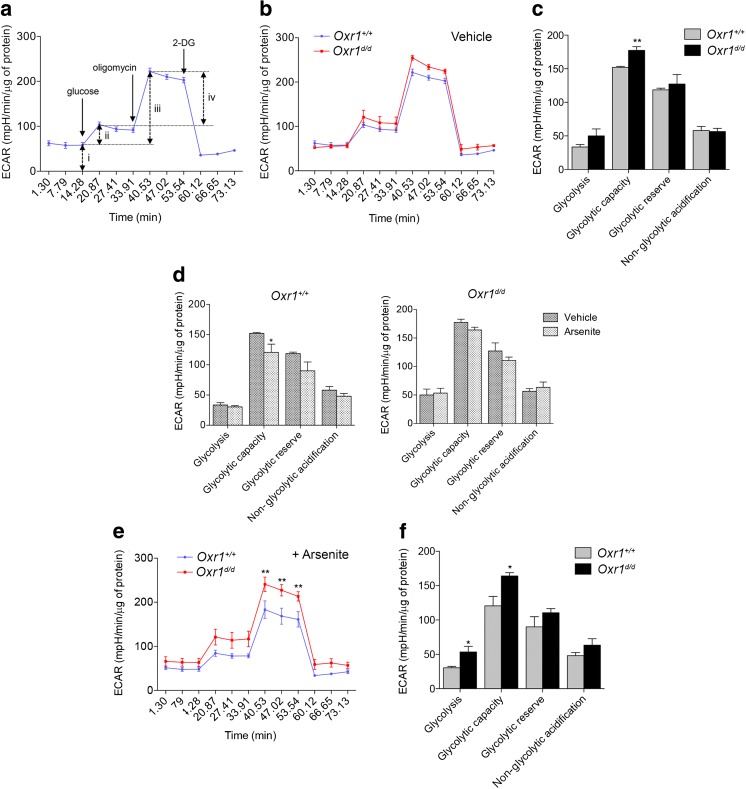
Glycolysis is altered in granule cells of Oxr1 knockout mice. **a** The extracellular acidification rate (ECAR) using the Seahorse glycolysis stress test assay; the timing of the compounds added to the assay medium are indicated (glucose to feed glycolysis, followed by oligomycin to inhibit mitochondrial ATP synthase and 2-deoxy-D-glucose (2-DG) to inhibit glycolysis) and the various parameters that can be calculated are represented by arrows (non-glycolytic acidification (i), glycolysis (ii), glycolytic capacity (iii), and glycolytic reserve (iv)). **b**–**f** ECAR assay data from primary CGCs from *Oxr1*^*d/d*^ and *Oxr1*^*+/+*^ mice in untreated (**b**–**d**) or arsenite-treated (**d**–**f**) conditions (*N* = 4 animals per group for all panels). For comparative purposes, panel **d** represents the data from panels **c** and **f**. Panels **b**, **e**: two-way ANOVA, panels **c**, **d**, **f**: *t* test; *p < 0.05, **p < 0.01

Next, we tested systematically which of the multiple aspects of Gpi1 function might be affected by Oxr1 in the brain. Gpi1 is a key glycolytic enzyme responsible for the non-committal interconversion between glucose-6-phosphate and fructose-6-phosphate [26]. To gain insight into whether loss of Oxr1 had any direct consequences for the glycolytic function of cells in the granule cell layer of the cerebellum, we determined the capacity of primary CGCs from *Oxr1*^*d/d*^ and wild-type controls to carry-out glycolysis when ATP production is inhibited. We measured glycolysis in real-time via the quantification of the extracellular acidification rate (ECAR) of the media in glucose-starved CGCs [35]. First, glucose was injected into the medium, followed by oligomycin to inhibit mitochondrial ATP synthase, and finally 2-deoxy-D-glucose (2-DG) to inhibit glycolysis; this allows glycolysis, glycolytic capacity, glycolytic reserve, and non-glycolytic acidification to be calculated [36]. A similar ECAR profile was observed in untreated CGCs from *Oxr1*^*d/d*^ mice compared to littermate controls (Fig. 3a–b), although glycolytic capacity was significantly increased in cells from mutant animals in response to mitochondrial inhibition (Fig. 3c). As Oxr1 plays a well-established role in protecting against oxidative stress-associated cell death, we next tested the ability of primary CGCs from *Oxr1*^*d/d*^ mice to regulate their glycolytic function under oxidative stress conditions. After pre-treatment with arsenite to induce oxidative stress, wild-type CGCs significantly reduced their glycolytic capacity as compared to untreated wild-type cells (Fig. 3d), while the other parameters were not significantly affected; this observation may represent an adaptive glycolytic response to prevent further production of ROS [11]. In contrast, when we compared the profile of cells from *Oxr1*^*d/d*^ mice in non-treated and in arsenite-treated conditions, no significant change in ECAR was observed (Fig. 3d). These data suggest that *Oxr1*^*d/d*^ cells, unlike control cells, do not adapt their glycolytic function when under oxidative stress. Furthermore, after arsenite treatment, both glycolysis and glycolytic capacity were significantly higher in CGCs from mutants compared to wild-type controls (Fig. 3e–f). In summary, these data provide further evidence that loss of Oxr1 leads to a significant disturbance of glycolytic metabolism that becomes more pronounced under conditions of oxidative stress.

### Gpi1 Modulates the Neuroprotective Properties of Oxr1

**Fig. 4:**
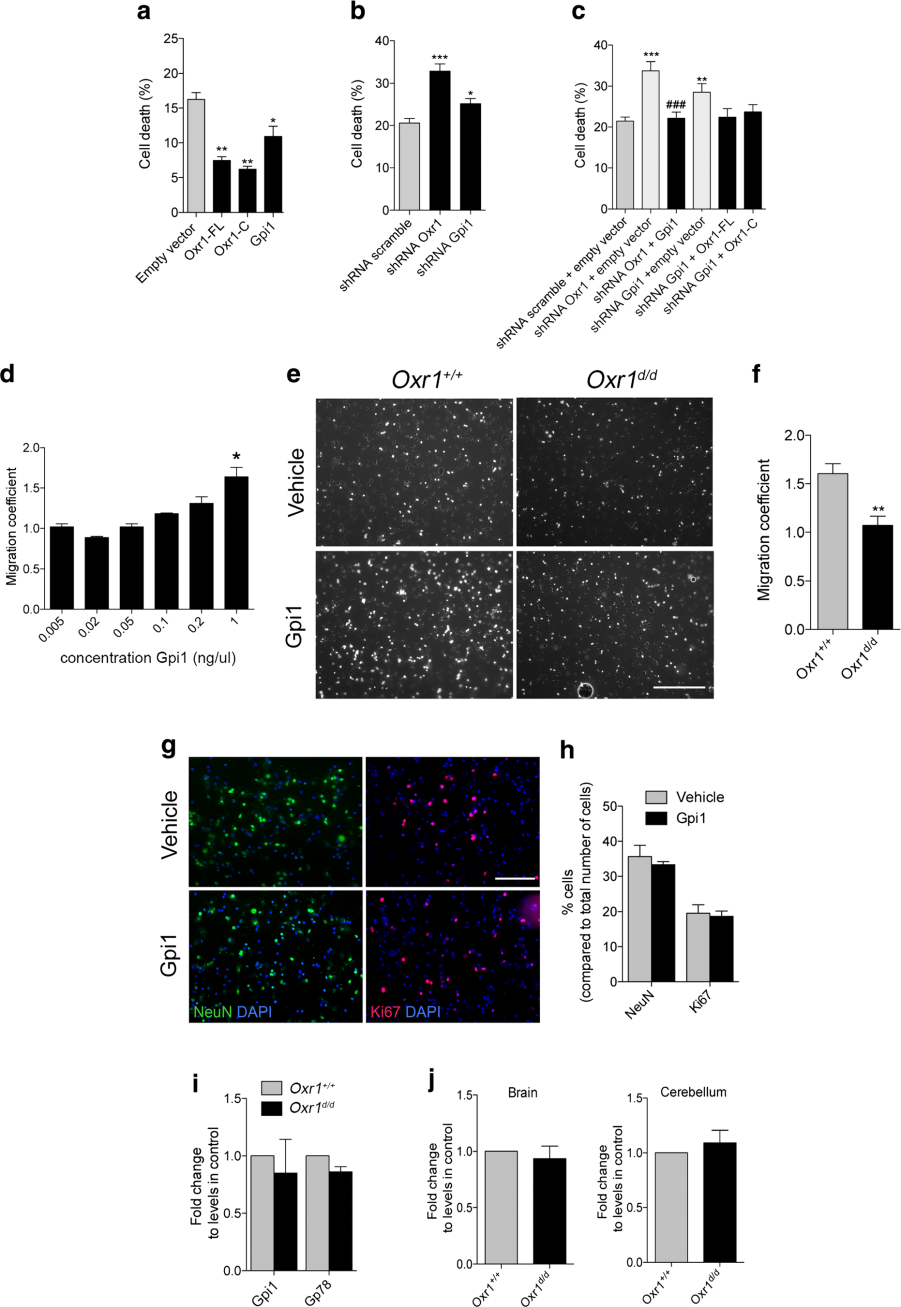
Gpi1 functions are modulated by Oxr1. **a** Over-expression of Gpi1, Oxr1-FL, and Oxr1-C in N2a cells treated with arsenite and quantification of pyknotic nuclei as a measure of cell death (*N* = 3 independent repeats). **b** Knockdown of *Oxr1* and *Gpi1* by shRNA in arsenite-treated N2a cells with quantification of pyknotic nuclei (*N* = 5 independent repeats). **c** Knockdown of *Oxr1* and *Gpi1* by shRNA together with either Gpi1 or Oxr1 over-expression, respectively, with quantification of the number of pyknotic nuclei (*N* > 5 independent repeats). **d** Wild-type CGCs cultured on Transwell inserts were treated with increasing concentrations of recombinant of Gpi1 and the number of cells having migrated through the insert were quantified (*N* = 2 repeats). **e** Representative images of CGC migration after 24-h treatment with recombinant Gpi1. Scale bar: 200 μm. **f** Quantification of migration coefficient in *Oxr1*^*d/d*^ and *Oxr1*^*+/+*^ cultures (*N* > 3 animals per group). **g**–**h** Relative composition of primary cultures treated with either vehicle or recombinant Gpi1 and quantified by immunocytochemistry using a neuronal marker (NeuN) and a marker for proliferating cells (Ki67). Scale bar: 100 μm. **i** Expression of *Gpi1* and its receptor, *Gp78*, in CGCs from *Oxr1*^*d/d*^ mice compared to *Oxr1*^*+/+*^ by qRT-PCR (*N* = 4 animals per group). **j** *Gp78* RNA expression levels in whole brain or cerebellum from P18 *Oxr1*^*d/d*^ mice compared to control littermates by qRT-PCR (*N* = 4–5 animals per group). Panels **a**–**d**: one-way ANOVA; **f**, **h**–**j**: *t* test; *p < 0.05, **p < 0.01, ***p < 0.001 as compared to shRNA scramble plus empty vector, shRNA scramble, empty vector, *Oxr1*^*+/+*^ or vehicle; ^###^*p* < 0.001 as compared to shRNA Oxr1 plus empty vector

Both Gpi1 and Oxr1 have been shown to possess neuroprotective properties [14, 37–39], thus we investigated next whether there was any correlation between the expression levels of Gpi1 and Oxr1 and their neuroprotective function. In a neuronal cell line treated with arsenite, cell death was quantified when Gpi1 or either isoform of Oxr1 was over-expressed. As previously described [16], both Oxr1-FL and Oxr1-C significantly reduced neuronal cell death induced by oxidative stress (Fig. 4a, Supplementary Fig. 3). Similarly, over-expression of Gpi1 led to a significant reduction in cell death (Fig. 4a); these data demonstrate that Gpi1, like Oxr1, can protect neurons under these conditions. Conversely, using shRNAs we confirmed that the reduction of Oxr1 expression led to a significant increase in neuronal cell death compared to a control scrambled shRNA vector in the same assay (Fig. 4b). Knockdown of Gpi1 also led to a significant increase in cell death compared to control cells, but to a lesser extent than Oxr1 knockdown (Fig. 4b, Supplementary Fig. 3). Importantly, when Oxr1 knockdown was combined with over-expression of Gpi1, there was no increase in cell death compared to cells transfected with control vectors (Fig. 4c, Supplementary Fig. 3). Furthermore, no change in cell death was observed when over-expression of either of the Oxr1 isoforms was combined with Gpi1 knockdown (Fig. 4c). Given that cell death induced by loss of either Gpi1 or Oxr1 can only be partially rescued by the over-expression of the other, this suggests that Gpi1 and Oxr1 are both important for neuroprotection against oxidative stress and that they rely on optimal expression levels of one another; thus providing further evidence for a functional interaction between the two proteins.

### Loss of Oxr1 Influences the Cytokine Function of Gpi1 in Neuronal Cells

In addition to its glycolytic function and as a growth factor promoting the survival of neurons, GPI can also act as a cytokine to stimulate cell proliferation and migration [27–29, 40]. First, we determined whether Gpi1 could induce migration of neuronal cells, as this has only been investigated in non-neuronal cells to date [41–43]. We quantified the migration of purified primary CGCs from wild-type mice treated for 24-h with a range of recombinant Gpi1 concentrations. We observed an increase in cell migration with increasing Gpi1 concentrations, confirming that Gpi1 can promote the migration of neuronal cells (Fig. 4d). Next, we went on to examine whether levels of Oxr1 would affect this specific aspect of Gpi1 function. We quantified the migration of cells from primary CGCs from *Oxr1*^*d/d*^ and littermate control mice after 24-h of treatment with the dose of recombinant Gpi1 protein determined previously (Fig. 4d–e). Interestingly, cells from *Oxr1*^*d/d*^ mice displayed a significantly reduced tendency to migrate after Gpi1 exposure compared to wild-type cultures (Fig. 4e–f). To control that the type and the properties of cells migrating in this assay were not affected by treatment with Gpi1, we confirmed that there was no significant difference in the proportion of neuronal NeuN-positive (approximately 35%) and proliferating Ki67-positive cells (approximately 19%) in the CGC cultures used and treated with either vehicle or Gpi1 (Fig. 4g–h). The extracellular receptor for Gpi1 is glycoprotein 78 (Gp78), which is highly expressed in neurons of the rodent brain [44, 45]. To confirm whether the reduced migration of *Oxr1*^*d/d*^ cells was not due to aberrant expression of *Gp78*, we quantified RNA levels of this gene in cultures used for the migration assay (Fig. 4i) as well as from cerebellar and the non-cerebellar brain tissue from *Oxr1*^*d/d*^ and control wild-type mice (Fig. 4j). No significant differences in *Gp78* expression were detected between the samples, suggesting that cells require Oxr1 to respond normally to secreted Gpi1. Thus, taken together, our data demonstrate an interaction between Gpi1 and Oxr1, which affects both neuroprotective function of Gpi1 and the cellular response to Gpi1 as a cytokine.

### Oxr1 Influences the Oligomerisation of Gpi1

**Fig. 5:**
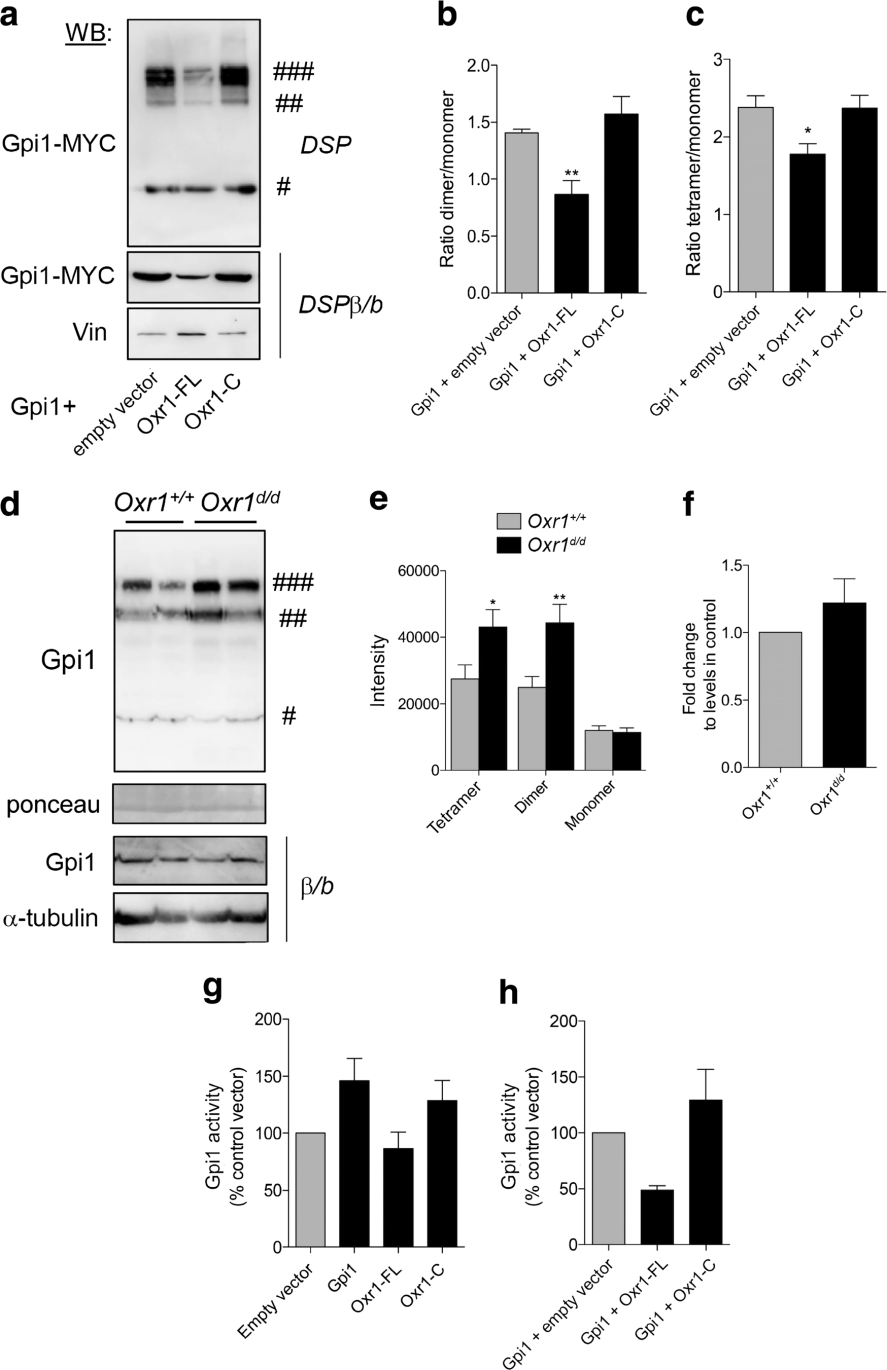
Oxr1 modulates Gpi1 oligomerisation. **a** Dimerisation of Gpi1 in cells co-transfected with Gpi1 and either an empty vector or full-length (Oxr1-FL) or short (Oxr1-C) Oxr1 isoforms. Cells were treated with a cross-linker (DSP) and proteins were extracted in PBS; the loading buffer did not contain any reducing β-mercaptoethanol and samples were not boiled (non-reducing conditions). As a control, protein extracts from cells treated with DSP were incubated with the reducing agent β-mercaptoethanol and boiled (DSPβ/b). Vinculin (Vin) levels were used to control for equivalent loading. **b**–**c** Quantification of the dimeric (**b**) or tetrameric (**c**) versus monomeric forms of Gpi1 (*N* = 6 independent repeats). **d**–**e** Western blot and quantification showing Gpi1 oligomerization in cerebellum from *Oxr1*^*d/d*^ and *Oxr1*^*+/+*^ mice from proteins extracted in PBS and non-reducing conditions. Ponceau staining was used to control for equal loading. As a control, protein extracts from the same preparations were incubated with the reducing agent β-mercaptoethanol and boiled (β/b). α-Tubulin levels were used to control for equivalent loading (*N* = 8 animals per group). **f** mRNA expression levels of *Gpi1* in the cerebellum of *Oxr1*^*+/+*^ or *Oxr1*^*d/d*^ mice by qRT-PCR (*N* = 4 animals per group). **g** Gpi1 activity in N2a cells transfected with the vectors indicated compared to an empty vector control (*N* = 3–5 independent repeats). **h** Gpi1 activity in N2a cells co-transfected with Gpi1 and either Oxr1-FL or Oxr1-C (*N* = 3 independent repeats). Panels **b**, **c**, **g**, **h**: one-way ANOVA; Panels **e**, f: t-test; *p < 0.05, **p < 0.01. Symbols #, ##, and ### represent Gpi1 monomer, dimer, and tetramer, respectively

GPI can be present as a monomer or as multimers in the cell, and the degree of oligomerisation is a key influence on its function; the dimeric form is composed of a large and a small domain, with the sugar binding site at the junction between these two domains responsible for its enzymatic activity [46–53]. The monomeric form of GPI, however, is considered to be involved primarily as a cytokine without enzymatic activity [28, 39, 52, 54, 55]. A tetrameric form of GPI has also been observed in vitro and in vivo*,* although the function of this species remains unknown [48, 54]. As we had already demonstrated that Gpi1 and Oxr1 can bind to one another, we next investigated whether this interaction would influence the oligomerisation of Gpi1. Initially, we co-transfected Gpi1 with either a control vector or Oxr1 in cells treated with the crosslinker and complex-stabilising dithiobis(succinimidylpropionate) (DSP) [54]. Cells over-expressing Gpi1 and treated with DSP presented three forms of Gpi1: monomeric, dimeric, and tetrameric (Fig. 5a); these dimeric and tetrameric forms were lost when protein extracts were incubated with β-mercaptoethanol and boiled to break any intra- or inter-molecular disulfide interactions (Fig. 5a) [54]. Interestingly, when co-transfected with Oxr1-FL, the ratio of dimeric and tetrameric forms over monomeric Gpi1 form was significantly reduced, while the levels of monomeric forms remained unchanged (Fig. 5). Such an effect was not observed when co-transfected with Oxr1-C (Fig. 5b–c). This suggests that Oxr1-FL can modulate Gpi1 oligomerisation in vitro.

Next, to investigate whether Oxr1 could modulate the level of Gpi1 oligomerisation in vivo, we extracted proteins from mouse cerebellar tissue in buffer conditions to preserve protein-protein interactions (PPIs). Interestingly, there was a significant increase in the absolute levels of Gpi1 tetramers and dimers in *Oxr1*^*d/d*^ cerebellum at the pre-symptomatic P18 timepoint compared to *Oxr1*^*+/+*^ controls as quantified by western blotting (Fig. 5d–e). To test whether the apparent changes in the proportion of the Gpi1 species were as a result of alterations at the transcriptional level, we also quantified *Gpi1* RNA. From the cerebella of mice from both genotypes, no significant difference in *Gpi1* expression was observed (Fig. 5f). Next, given that Oxr1 changes the proportion of Gpi1 dimerization—which is essential for Gpi1 glycolytic activity—we wanted to test whether an increase in Oxr1 may affect Gpi1 activity in vitro. Cells over-expressing either Gpi1 or Oxr1-C showed a slight increase in Gpi1 activity as compared to cells transfected with a control empty vector (Fig. 5g). However, when co-transfected with Gpi1, Oxr1-FL reduced Gpi1 activity approximately two-fold as compared to cells co-expressing Gpi1 with an empty vector or Oxr1-C (Fig. 5h). These data suggest that Oxr1-FL has a propensity to modulate Gpi1 dimerisation levels and is also able to affect Gpi1 activity.

### Disease-Causing Mutations in Gpi1 Influence Oxr1 Binding

**Fig. 6:**
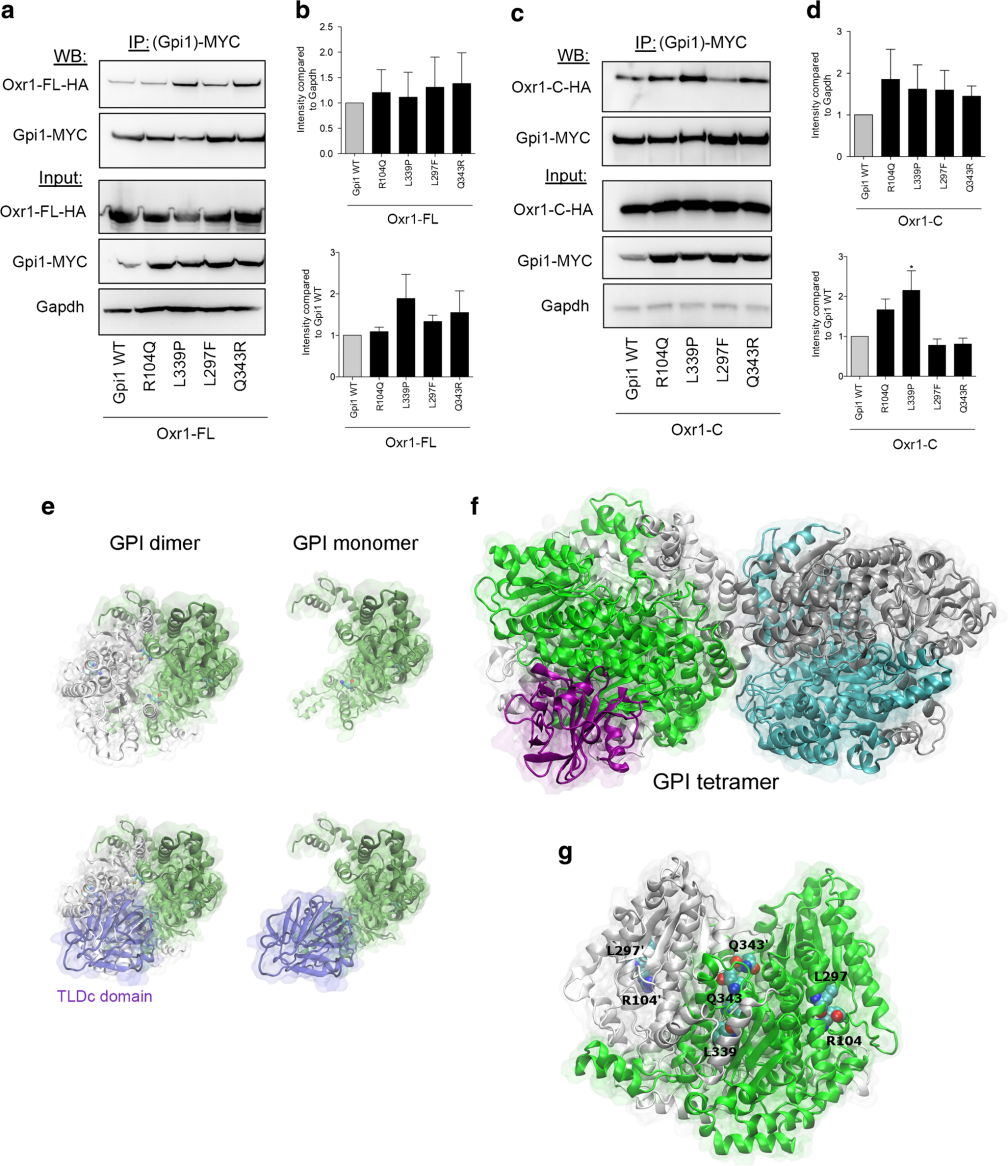
Oxr1 binding affinity to Gpi1 is influenced by disease associated-mutations. **a**–**d** Co-immunoprecipitations using an anti-MYC antibody and direct protein extracts in cells co-expressing MYC-tagged Gpi1 (wild-type (WT) or mutant constructs) with HA-tagged Oxr1-FL (panels **a**–**b** *N* = 3 independent repeats) or Oxr1-C (panels **c**–**d** *N* = 4 independent repeats). **e**–**f** Predictions of the monomeric (**e**), dimeric (**e**), and tetrameric (**f**) forms of mouse Gpi1 interacting with the TLDc domain. **g** Representation of the Gpi1 dimer with the positions of the key residues mutated and investigated highlighted. Panels **b**, **d**: one-way ANOVA; *p < 0.05

GPI deficiency caused by genetic mutations is a known cause of hemolytic anaemia [56–72]; a subset of these mutations is also associated with neurological symptoms, including intellectual disability and neuromuscular dysfunction [30–34]. The reason for this disease spectrum remains unclear; however, predictions based on the three-dimensional structure of GPI suggest that certain mutations may be more likely to affect either the glycolytic or cytokine activities of the protein [51]. Thus, we investigated next whether the functional interaction between Oxr1 and Gpi1 we discovered would be influenced by conserved pathogenic GPI mutations. We chose to test a set of mutations spanning the coding sequence that have been associated specifically with either non-neurological (Q343R) [60] or neurological (R104Q, L297F, L339P) symptoms [30, 33]. Using co-immunoprecipitation in cells expressing either wild-type or mutant Gpi1 with Oxr1-FL, we showed that these four particular mutations do not appear to affect the affinity between the two proteins or their expression levels (Fig. 6a–b). As we have demonstrated that Oxr1-C also binds to Gpi1, the same set of co-immunoprecipitations was also carried out with this short isoform (Fig. 6c–d). Interestingly, the L339P Gpi1 mutation showed a significant increase in affinity for Oxr1-C compared to wild-type Gpi1, although the level of expression was not altered (Fig. 6d).

To gain a better understanding of the binding between Oxr1 and Gpi1, we used in silico modelling of the PPI between these two proteins. In the absence of an atomically-detailed structure of Oxr1, we produced a model of the TLDc domain of mouse Oxr1 using the 0.97-Å resolution crystal structure of the zebrafish TLDc domain from Oxr2 as a template [73]. The resulting model was then used in combination with the structure of mouse Gpi1 as input to the protein-protein docking ClusPro server to generate multiple docking models. As the Oxr1-C isoform does not seem to significantly affect the dimerisation levels of Gpi1 (Fig. 5), we studied the PPI of the Oxr1 TLDc domain with Gpi1 in its monomeric, dimeric, or tetrameric form (Fig. 6e–f). An exhaustive rigid-body protein docking was performed to identify the most favourable interaction interface between the available TLDc domain structure and Gpi1. Accordingly, the most populated cluster showed that the TLDc domain overlaps the second Gpi1 protein of the Gpi1 dimer (Fig. 6e), suggesting that both proteins share a common protein-protein interface.

**Fig. 7:**
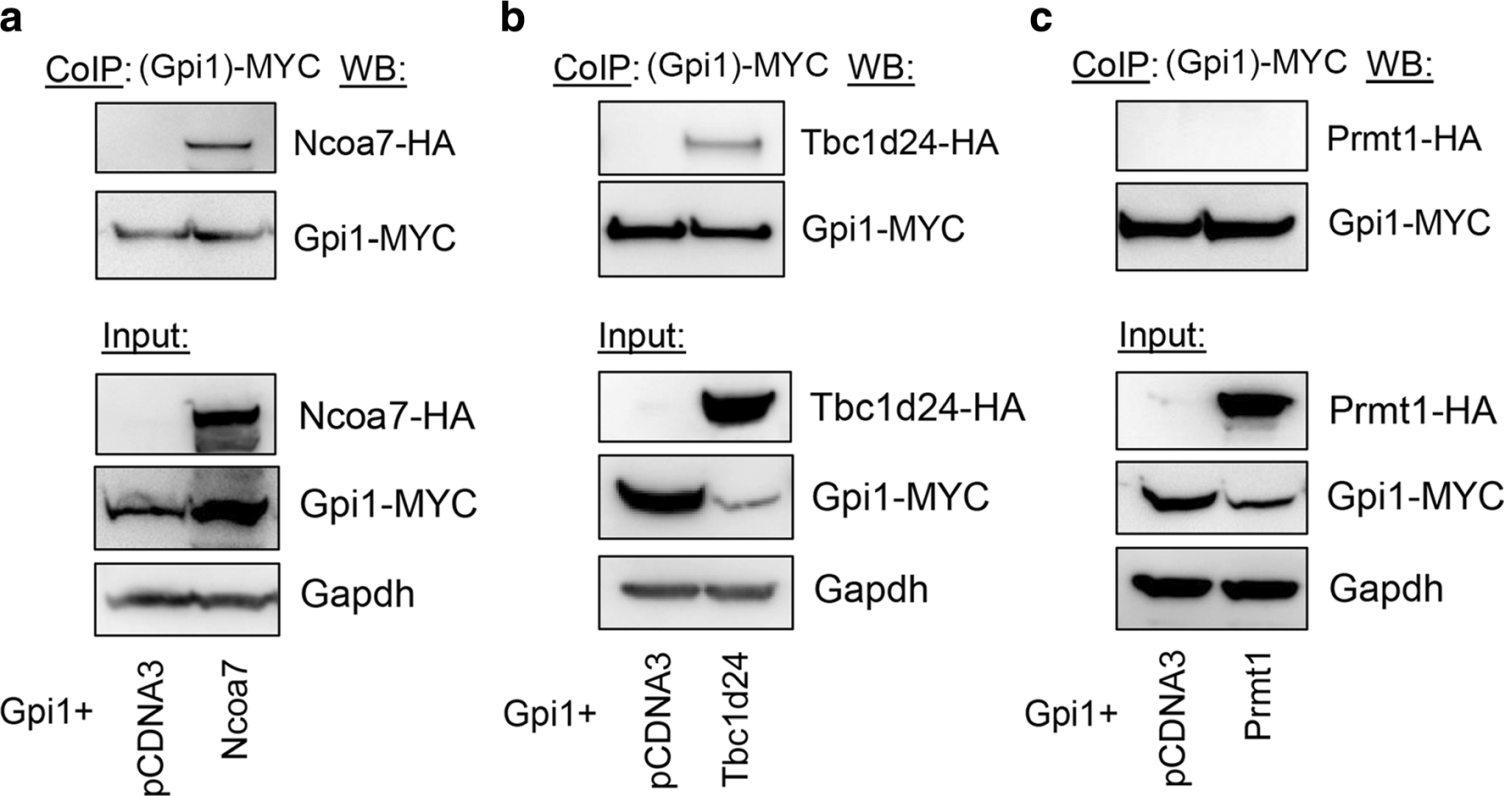
Ncoa7 and Tbc1d24 are protein interactors of Gpi1. Co-immunoprecipitation in HeLa cells co-transfected with MYC-tagged Gpi1 and either a control vector or HA-tagged Ncoa7, Tbc1d24, or an unrelated protein Prmt1 as a negative control.

Interestingly, the most populated cluster with the largest number of low-energy conformations—and therefore the most energetically favourable model—shows the TLDc domain binding site overlapping the key neurodegenerative disease mutation residues R104 and L297 (Fig. 6g); this interaction also involves several charged residues in the TLDc domain, including R126, D156, and E190. Of note, all three of these amino acids are conserved in the TLDc domain of human and mouse NCOA7, the protein most closely related to Oxr1, and two are conserved in human and mouse TBC1D24 [12, 16, 18]. Therefore, we tested the binding of mouse Ncoa7 and Tbc1d24 to Gpi1 and found that the interaction could be detected reproducibly and specifically (Fig. 7). Together, these findings provide further evidence that the TLDc domain is key to the Gpi1 interaction; furthermore, as the loss of Oxr1 leads to a global increase of oligomeric Gpi1 (Fig. 5), we could also hypothesise that this predicted binding site is a new interface that modulates transition of Gpi1 between monomeric and multimeric forms.

## Discussion

We have shown here that Oxr1 possess an unexpected function as a regulator of glucose metabolism, in particular under oxidative stress conditions. This new role for Oxr1 is carried out—at least in part—via a direct interaction with Gpi1. In addition, we reveal that Oxr1 is the first protein described that is able to modulate both the neuroprotective and cytokine properties of the multi-functional Gpi1 protein.

Using the *Oxr1* knockout mouse model, we have identified a new mechanistic link between glucose metabolism and neurodegeneration in a defined region of the brain. It is also noteworthy that the dysregulation of metabolite levels we observed downstream of Gpi1 occurs at the pre-symptomatic stage, suggesting that this metabolic disturbance plays a role in the cerebellar-specific pattern of cell death. This apparent selective vulnerability of the cerebellum has also begun to be investigated in disorders, such as spinocerebellar ataxia (SCA) using quantitative metabolite profiling. For example, in two mouse models expressing a mutated SCA1 (Ataxin-1) gene (82Q and 154Q/2Q), an increasing level of glutamine was observed consistently in this region by magnetic resonance spectroscopy (MRS) [74, 75]. Increased glutamine was also detected in the cerebellum of SCA1 patients using the same non-invasive method [76]. Importantly, there was no correlation between glutamine levels and ataxia scores in symptomatic patients or pathology scores in symptomatic SCA1 mutant mice, suggesting that the accumulation of this specific metabolite was a biochemical marker of the pre-symptomatic disease state [74, 76]. More recently, proteomic profiling of the cerebellum from homozygous Ataxin-1 knockout mice at a pre-symptomatic stage revealed expression changes in a range of key bioenergetic pathways, including Gpi1 and other glycolytic enzymes [77]. Although the molecular mechanisms linking mutant Ataxin-1 to these pathways are unknown, alongside our new data, these findings suggest that metabolic disruption may be a key feature of cerebellar disorders. Furthermore, levels of glucose and glycogen—the main form of glucose storage in the brain—are higher in the cerebellum than other brain regions, supporting the hypothesis that the cerebellum is distinct from other brain regions regarding its energy buffering capacity [78]. Whether these data reflect the complex network connectivity requirements of the densely packed granule cell layer is still not entirely clear [22]; in particular, as the importance of glucose metabolism for non-neuronal—as well as neuronal—cell populations in the CNS is only just beginning to be understood. For example, astrocytes play an essential role in the uptake of glucose, and the availability of metabolites can have a major influence on astrocyte and glial cell function [79–81]. Given that we have demonstrated previously that over-expression of Oxr1 in vivo can delay the neuroinflammatory response in a model of ALS [17], the relationship between glycolytic pathways and Oxr1 function in non-neuronal cells warrants further investigation in the future.

Our in vivo data suggests that loss of Oxr1 increases Gpi1 affinity for its substrates and/or favours the reaction converting glucose-6-phosphate to all three downstream metabolic pathways (glucuronic acid pathway, glycolysis, and oxidative PPP). PPP in particular shows higher levels of intermediates, particularly in the oxidative part of the pathway. However, we did not detect a significant increase in Gpi1 activity in whole cerebella from *Oxr1*^*d/d*^ mice. This could be explained by the fact that Gpi1 activity is also inhibited by the glycolytic or PPP intermediates, for example 6-phospho-gluconic acid [82], which is significantly increased in cerebella of *Oxr1*^*d/d*^ mutants compared to controls in our study.

Our data in *Oxr1* knockout brain tissue indicates that Oxr1 regulates the function of Gpi1 by modulating the ratio of monomeric to oligomeric forms and thus potentially favours Gpi1 activity as a cytokine over its glycolytic role. Thus, to understand the multiple aspects of Gpi1 functional regulation, one needs to consider not only the expression of the protein but also the degree of oligomerisation. Indeed, it has been demonstrated using deletion mutants around a conserved cysteine motif that both the monomeric cytokine function and ligand-receptor binding to Gp78 are independent of the enzymatic activity of GPI as a dimer [55]. Therefore, we used a combination of molecular and in silico modelling approaches to identify potential binding sites between Gpi1 and Oxr1 and to study the effect of disease-associated mutations on binding affinity. Clinically, GPI mutations significantly reduce the catalytic activity of the protein; these measurements are made typically from patients’ erythrocytes, although much of the molecular data linking phenotype and genotype are based-around the physiochemical properties of the GPI protein, such as in vitro thermostability or electrophoretic mobility [30–32, 34, 59–65, 67, 69, 72, 83–85]. In addition, the molecular detail of enzymatic dysfunction can be confounded by the presence of compound heterozygous GPI mutations, where an unknown complement of mutant catalytic dimers or heterodimers will form in vivo [30, 33, 34, 60, 62, 63, 65–67, 70]. A previous study examined mutants in the context of the GPI three-dimensional data and classified them in distinct classes: those that alter GPI structure and those that disrupt either its oligomerization or active site [51]; thus it was hypothesised that mutations influencing protein folding would affect both the enzymatic and neurotrophic activities of GPI leading to haemolytic and neurological symptoms in patients, while mutations affecting the active site would disrupt the enzymatic activity alone [30, 51, 70, 86]. Here, we demonstrated that there were limited effects on binding affinity between Oxr1 and the selected pathogenic Gpi1 mutations, although the shortest Oxr1-C isoform interacted with greater affinity to the L339P mutant, a substitution that causes anaemia with neuromuscular involvement when combined with a second H20P mutant GPI allele [30]. A study on recombinant proteins has demonstrated that the L339P substitution significantly reduces the stability and catalytic activity of GPI, and it was proposed that the new proline residue would disrupt multiple internal hydrophobic bonds [85]. How Oxr1 functions in the context of GPI deficiency is yet to be investigated, although it is interesting that we have previously shown the utility of Oxr1 over-expression in delaying neuromuscular-associated phenotypes [17].

We showed previously that loss of *Oxr1* led to an increase in apoptotic cell death in the granule cell layer of the cerebellum from P19 as well as in cultured primary CGCs under oxidative stress [14]. Here, we investigated glycolysis under oxidative stress conditions and discovered that CGCs lacking *Oxr1* do not reduce their glycolytic rate under oxidative stress, as would be expected in wild-type cells to reduce the over-production of ROS [11]. Thus, we can hypothesise that Oxr1 carries out its neuroprotective function, at least in part, by modulating Gpi1 activity in order to inhibit the glycolytic pathway and dampen ROS production. However, this functional association is likely to be more complex; indeed, we showed here that both the full-length and shortest TLDc domain-containing isoforms of Oxr1 may influence Gpi1 function differently. These data are particularly interesting, as Oxr1 isoforms have been reported to be differentially induced under sustained oxidative stress conditions [15, 87, 88]; together these data suggest a model whereby Oxr1-FL enhances initially the ratio of monomeric to high molecular weight species of Gpi1, while the glycolytic dimeric forms are stabilised subsequently by Oxr1-C during oxidative stress. Currently, however, it is unclear whether the binding of one Oxr1 isoform to Gpi1 would prevent any interaction with another Oxr1 isoform. In addition, we note that our modulation of Gpi1 expression does not appear to impact cell death as strongly as the knockdown or over-expression of Oxr1. These data could suggest that—although Oxr1 binding to Gpi1 modulates glycolytic function and consequently ROS production—this interaction influences another feature of Oxr1 that plays a more direct role in cell survival; for example, by impacting other PPIs [15]. Alternatively, these findings may relate to the relative knockdown or over-expression efficiencies and mRNA levels obtained, or to unknown thresholds of expression that are required to influence the particular pathways we have studied.

Our data also indicate that primary neurons lacking Oxr1 do not respond normally to stimulation by Gpi1 acting as an extracellular chemokine. This particular function of GPI is known to be signalled via an interaction with its extracellular receptor (GP78). Therefore, in our particular assays, the functional interaction between Oxr1 and Gpi1 may be indirect. However, a study has demonstrated that extracellular monomeric GPI can be internalised by endocytosis [89], thus facilitating potential downstream interactions with proteins that may influence cell migration. Further studies will be required to examine this hypothesis.

Finally, in-line with the apparent importance of the TLDc domain for this GPI interaction, we identified the related TLDc proteins Ncoa7 and Tbc1d24 as additional novel binding partners for Gpi1, as predicted by conserved putative binding residues. This provides new molecular evidence that there is functional overlap between TLDc proteins in the brain. Moreover, these data may relate to specific pattern of cell death in the *Oxr1*^*d/d*^ cerebellum, a region where there is limited concordant expression of *Oxr1* and *Ncoa7* [16, 90]. In other brain areas where no cell death occurs, such as the cerebral cortex and hippocampus, all three genes are highly co-expressed, suggesting that Ncoa7 and Tbc1d24 may be able to compensate functionally for loss of Oxr1 [16, 90].

The disruption of energy homeostasis is becoming a more common theme in neurological disease with the discovery that abnormal metabolic function occurs both prior to, and as part of, the pathological process. Here, we have shown that glycolytic imbalance is a feature of cerebellar neurodegeneration in *Oxr1* knockout mice, and that multiple aspects of Gpi1 function can be modulated by the Oxr1 protein itself. There is still much to learn regarding the role of GPI in the nervous system; indeed, it is noteworthy that knockdown of GPI in neuronal cells leads to caspase-dependent apoptotic cell death [91], and more specifically, to an increased neurotoxicity in primary cortical neurons via the accumulation of insoluble alpha-synuclein [38]. Whether such findings relate to GPI oligomerisation or its receptor is still unclear [92], yet our identification of Oxr1 as a new regulator of GPI may shed more light on the complexities of this important moonlighting protein.

## Materials and Methods

### Animal Experimentation

Constitutive Oxr1 knockout (*Oxr1*^*d/d*^) mice were derived from the previously described *Oxr1*^tm1a(EUCOMM)Wtsi^ line, in which a LacZ reporter was inserted in the TLDc domain, while two adjacent exons are flanked by loxp sites (International Knock-out Mouse Consortium (IKMC) program, IKMC project 84243) [16, 18]. *Oxr1*^*tm1a*^ mice were first crossed with a ubiquitous FlpE recombinase-expressing line to remove the neomycin selection cassette in the construct, followed by removal of the FlpE transgene by backcrossing to C57BL6/J mice. These *Oxr1*^*tm1*c^ allele-carrying mice were then crossed to a ubiquitous cre-recombinase expressing line to generate the *Oxr1*^*tm1d*^ allele, followed by removal of the cre transgene by backcrossing to C57BL6/J and subsequent backcrossing to C57BL6/J for eight generations. Homozygous *Oxr1*^*d/d*^ knockout animals were generated by intercrossing mice heterozygous for the *Oxr1*^*tm1d*^ allele. Expression of the *Oxr1*^*tm1d*^ allele results in the removal of two exons in the TLDc domain and the introduction of a premature stop codon, removing the terminal 101 amino acids of all Oxr1 protein isoforms (Fig. 1 and Supplementary Fig. 1). All experiments were conducted in adherence to the guidelines set forth by the UK Home Office regulations, and with the approval of the University of Oxford Ethical Review Panel.

### Cell Culture, Transfection, and Treatment

Neuronal Neuro2a (N2a) and HeLa cells were cultures in Dulbecco’s modified Eagle’s medium (DMEM) supplemented with glutamax, 1% penicillin-streptomycin, and 10% fetal calf serum (all Gibco). Cells were co-transfected with Fugene 6 (Promega) for 48 h as per the manufacturer’s protocol. shRNA constructs were from Sigma (#11857 for Gpi1 and as previously described for Oxr1 [14]). For neurite growth assay, medium was replaced with serum-free medium when transfecting and incubated for 48 h. For cell death assay, cells were treated with 250-μM arsenite (Sigma) for 4 h. Cells were fixed with 4% paraformaldehyde for 10 min at room temperature, washed with PBS (Sigma) twice, and blocked with blocking buffer (5% goat serum (VectorLab), 0.5% Triton X-100 (Sigma)) for 1 h at room temperature. Neurites and pyknotic nuclei were visualised with NF200 antibody (Sigma N4142) and DAPI staining (Vectorlabs), respectively.

### Granule Cell Culture and Cell Mobility Assay

Cerebella from P7 pups were dissected in cold HBSS (Gibco), the meninges removed and were incubated in tryspin (Gibco) for 15 min at 37 °C. The reaction was then stopped by adding trypsin inhibitor (Life Technologies) and incubating at room temperature for 5 min. Cerebella were triturated and purified on an Optiprep gradient [93]. Cells were plated in DMEM (Gibco) supplemented with 0.5-mM glutamine (Gibco). Cell migration was assessed using an 8.0-μm pore polycarbonate membrane Transwell inserts (Corning Life Sciences, Acton, MA). The lower surface of the insert was pre-coated with laminin (Sigma). Granule cell suspensions (20 × 10^4^ cells) were added to the upper compartment, and 600-μl culture medium was added to the lower compartment with recombinant Gpi1 (Abcam ab87625) or vehicle (water). After a 24-h incubation at 37 °C, the membranes were fixed with 4% paraformaldehyde for 10 min at room temperature and washed with PBS. Cells on the upper surface of the membrane were swiped with cotton swabs and cells that invaded through the membrane to the under membrane were visualised by DAPI staining (VectorLab) and counted using a fluorescence microscope (Leica).

### Immunohistochemistry

TUNEL staining on frozen (15 μm) sections was carried out using the In Situ Cell Death kit as per the manufacturer’s protocol (Roche). TUNEL-positive cells from five whole cerebellar sections from the midline of the brain at 60-μM intervals were counted from each individual mouse.

### Co-Immunoprecipitation, Protein Dimerization, and Western Blotting

To quantify the binding of Gpi1 and Oxr1 in cells, HeLa cells over-expressing Gpi1 and Oxr1 were washed once in cold PBS and lysed with cold standard RIPA buffer (50-mM Tris pH 7.5, 150-mM NaCl, 0.1% SDS, 0.5% sodium deoxycholate, 1% Triton X-100, all from Sigma) supplemented with protease and phosphatase inhibitor cocktail (Cell Signaling) by passing the cells through a 23-gauge needle (> 10 strokes). After 30-min incubation on ice and centrifugation at maximum speed for 20 min at 4 °C, protein content was quantified by BCA assay (Thermo Scientific). MYC-tagged proteins were pulled down using 50 μl of anti-MYC EZview beads (Sigma) and incubated overnight at 4 °C. Immunoprecipitated proteins were washed three times in RIPA buffer before being boiled in NuPAGE loading buffer (Life Technologies) supplemented with β-mercaptoethanol (Sigma). Proteins were run on pre-cast NuPAGE Bis-Tris gels (Life Technologies) and transferred as per the manufacturer’s protocol.

To assess the level of oligomerisation of Gpi1, HeLa cells were co-transfected with Gpi1 and an empty vector or with Oxr1-FL or Oxr1-C. After 2-day transfection, cells were washed once with cold PBS and scraped from the dish surface in PBS supplemented with either 1-mM dithiobis(succinimidyl propionate) (DSP, Thermo Scientific) and lysed by passing through a 23-gauge needle and incubated at room temperature for 30 min. The reaction was then quenched by adding Tris pH 7.8 to reach 50-mM final concentration and incubated at room temperature for 15 min. Cell extracts were clarified by centrifugation at maximum speed at 4 °C for 15 min. For oligomerisation in tissue, mouse cerebella were homogenised in PBS using a tissue Precellys homogeniser (Bertin Corp.) and centrifuged for 30 min at 4 °C at maximum speed. Protein extract was quantified by BCA assay (Thermo Scientific). An equal amount of proteins in Laemmli buffer (Biorad) supplemented with or without β-mercaptoethanol and boiling as indicated in the figure legends was run on 8% SDS-PAGE gels. Primary antibodies used for western blotting were as follows: Oxr1 (antiserum produced in the laboratory), HA (Roche 11867423001 and Sigma H6908), MYC (Sigma M4439), GAPDH (Covance MMS-580S), Vinculin (Abcam ab73412), Gpi1 (Abcam ab66340), and α-tubulin (Sigma T5168). Secondary HRP antibodies were from Invitrogen. Antibody signal was detected using ECL or ECL prime reagent (GE). The signal was detected using an ImageQuant LAS4000 (GE Healthcare).

### RNA Extraction and Quantitative Real-Time PCR (qRT-PCR)

RNA was extracted from cells or mouse tissue using an RNeasy mini kit (Qiagen). RNA was reversed transcribed (RevertAid, Thermo Scientific) and qRT-PCR was performed in an ABI PRISM 7000 sequence detection system (Applied Biosystems) using SYBR green PCR master mix (Applied Biosystems) with primer sequences are shown in Table S2. The reference gene *Gapdh *was used as an internal normalising control. The ΔΔCt method was used to calculate the fold change as compared to control samples.

### Glucose-6-Phosphate Isomerase Glycolytic Activity

Gpi1 activity was measured in N2a cells transfected for 48 h or in whole brain or cerebellum using the colorimetric glucose-6-phosphate isomerase activity assay kit (Abcam), following the manufacturer’s protocol. Cells and tissue were homogenised in cold assay buffer supplemented with protease inhibitors provided. After centrifugation at 12,000 rpm for 10 min at 4 °C, cleared protein samples were measured by BCA assay (Thermo Scientific) and equal amount of protein was used per reaction. Immediately after the reaction mix was added to the samples, absorbance was measured at 450 nm every 5 min for 3 h on a Fluostar Omega plate reader (BMG LabTech). A standard curve of NADH was generated in parallel using the NADH provided. After subtracting the background reading to all values, the Gpi1 activity from the sample was calculated by applying the difference in absorbance between the two extreme time points of the linear range for the sample to the NADH standard curve. This value was then corrected by the reaction time, volume per well, and the dilution factor.

### Quantification of Fructose-6-Phosphate (F6P)

Mouse cerebella were homogenised in ice-cold PBS using a Precellys homogeniser (Bertin Corp.) and extracts were clarified by centrifuged for 10 min at maximum speed at 4 °C. Protein amounts were quantified by BCA assay (Thermo Scientific) and equal amount of material was then used and deproteinizated using a deproteinizing sample preparation kit (ab204708). A standard curve was generated using the F6P provided. Once the reaction master mix was added to the samples, fluorescence was read on a Fluostar Omega plate reader (Ex/Em = 544/590) (BMG LabTech). After subtracting the background reading to all values, the concentration of F6P in the sample was equals to the amount of F6P in the sample from the standard curve corrected for the sample volume added per reaction per well and the dilution factor.

### Glucose Stress Assay

To measure glycolysis through proton production [36], a Seahorse extracellular flux (XF) analyser (Seahorse Biosciences) was used as per the manufacturer’s protocol. Briefly, non-purified granule cells were prepared as described above and were grown in DMEM supplemented with 0.5-mM L-glutamine and 10% fetal calf serum (Gibco) on 96-well plates pre-treated with poly-ornithine (Sigma). The day after splitting, cells were treated with 0.01-mM cytosine-1-β-D-arabinofuranoside (Sigma) and cultured for 7 days. On the day of the assay, cells were treated with vehicle or 25-μM arsenite for 4 h before being transferred to minimum medium (Seahorse XF glycolysis stress test buffer supplemented with 2-mM L-glutamine Gibco, pH 7.4) for 1 h. To quantify glycolysis levels, the glycolysis stress test assay is performed by first injecting glucose (to feed glycolysis), then oligomycin (to drive glycolysis), and finally (to inhibit glycolysis). Glycolysis is determined through measurements of the extracellular acidification rate (ECAR) of the surrounding media in which glucose, oligomycin, and 2-deoxyglucose (2-DG) are sequentially added. The ECAR values were normalised to the protein level per well, as determined by BCA assay at the end of the run.

### Sample Preparation for Metabolomics Profiling

Whole cerebella were homogenised in 300-μl ice-cold 80% methanol in a homogeniser vial (Precellys). After centrifugation at maximum speed for 20 min at 4 °C, protein content was quantified by BCA assay, and equal amount of sample was filtered through a 10-kD molecular weight cut-off filters (Amicon) to remove soluble proteins from the metabolite solution.

### Metabolomic Profiling by Ion Chromatography-Tandem Mass Spectrometry (IC-MS/MS)

Each sample was analysed by IC-MS/MS using an ICS-5000+ ion chromatography system coupled directly to a Q-Exactive HF Hybrid Quadrupole-Orbitrap mass spectrometer with a HESI II electrospray ionisation source (Thermo Scientific) [23–25]. The ion exchange chromatography system incorporated an electrolytic anion generator (KOH), which was programmed to produce a OH^−^ gradient over 37 min. An inline electrolytic suppressor removed OH^−^ ions and cations from the post-column eluent stream prior to MS analysis (Thermo Scientific Dionex AERS 500). A 10-μL partial loop injection was used for all analyses, and the chromatographic separation was performed using a Thermo Scientific Dionex IonPac AS11-HC 2 × 250 mm, 4-μm particle size column with a Dionex Ionpac AG11-HC 4-μm 2 × 50 guard column inline. The IC flow rate was 0.250 mL/min. The total run time was 37 min and the hydroxide ion gradient comprised as follows: 0 min, 0 mM; 1 min, 0 mM; 15 min, 60 mM; 25 min, 100 mM; 30 min, 100 mM; 30.1 min, 0 mM; and 37 min, 0 mM. Analysis was performed in negative ion mode using a scan-range from *m*/*z* 60–900 and resolution set to 70,000. The tune file source parameters were set as follows: sheath gas flow 60 mL/min; Aux gas flow 20 mL/min; spray voltage 3.6v; capillary temperature 320 °C; S-lens RF value 70; heater temperature 350 °C. AGC target was set to 1e6v ions and the Max IT value was 250 ms. The column temperature was kept at 30 °C throughout the experiment. Full scan data were acquired in continuum mode.

### Processing and Data Analysis

Raw data files were processed using ProgenesisQI (Waters) [23–25]. This process included alignment of retention times, peak picking by identification of the presence of natural abundance isotope peaks, characterising multiple adducts forms and identification of metabolites using an in house database. Retention times, accurate mass values, relative isotope abundances, and fragmentation patterns were compared between authentic standards and the samples measured. Identifications were accepted only when the following criteria were met: < 5-ppm differences between measured and theoretical mass (based on chemical formula), < 30-s differences between authentic standard and analyte retention times, and isotope peak abundance measurements for analytes were > 90% matched to the theoretical value generated from the chemical formula. Where measured, fragmentation patterns were matched to the base peak and two additional peak matches in the MS/MS spectrum to within 12 ppm. The top 10 data directed fragmentation method was not always able to provide fragment ions for all ions measured in the MS 1 spectrum. PCA was performed using the capability for this output in Progenesis QI and SIMCA 14 (Umetrics). This analysis is “un-supervised” and retains no “knowledge” of the experimental groups themselves; so, this approach is useful for confirming compound abundance similarities and differences between groups of samples and for identifying any sample outliers. Fold change, % coefficient of variation (%CV) and *p* values were generated automatically in Progenesis QI and verified manually using a normalised abundance output and Excel. Heat maps were generated manually using the verified fold-change output.

### Modelling

A model of the mouse TLDc domain was generated with Modeler [94] using the structure of the zebrafish TLDc (PDB: 4ACJ) as a template [73]. The sequence alignment between the two proteins was performed with Clustal Omega [95] achieving a 62% sequence identity. The resulting structure was subsequently used to predict its interface with Gpi1 (PDB: 1U0E) using the Cluspro server [53, 96]. An exhaustive rigid-body protein docking was performed using Cluspro followed by a clustering of the results, which are also energy minimised. As the size of each cluster is proportional to its probability of being the lowest energy configuration [96], the most populated cluster is the most likely to represent the most favourable interaction interface between two proteins. The same interface was reproduced using the three scoring schemes implemented in Cluspro (i.e. balanced, electrostatic-favoured, and hydrophobic-favoured) as well as the “others mode,” which is designed to fit protein interactions differing from the enzyme-inhibitor and multi-subunit proteins used to parameterize the scoring schemes above. In all cases, the most populated cluster was at least 2 to 4 times larger than the second most populated, as indicated to increase the level of certainty 5.

## Electronic Supplementary Material

Supplementary Table 1 List of metabolites identified as different in the cerebella of *Oxr1*^*d/d*^ as compared to *Oxr1*^*−/−*^ mice. Fold change, % coefficient of variation (%CV), and *p* values were generated automatically in Progenesis QI and verified manually using a normalised abundance output and exported in Excel. (XLSX 28 kb)

Supplementary Table 2 Sequences of primers used for qRT-PCR. (XLSX 13 kb)

Supplementary Fig. 1 Schematic of mouse Oxr1 isoforms based on the UCSC Genome Browser (GRCm38/mm10) showing the range of annotated full-length (FL) isoforms with alternative start sites and the shortest isoform (**c**). Coding exons (white), UTRs (grey), an alternatively-spliced exon (yellow), and the TLDc domain (blue) are shown. Not to scale. The Oxr1 knockout allele (tm1d) used in this study is also indicated that truncates all of the above isoforms. This allele was generated by from the corresponding tmlc allele with loxp sites (red arrows) flanking two coding exons in the TLDc domain. (PNG 41 kb)

High-resolution image (TIF 136 kb)

Supplementary Fig. 2 Pathway analysis of the metabolites dysregulated in the *Oxr1*^*d/d*^ cerebellum. These metabolites were mainly from the glycolysis, pentose phosphate pathway, and the TCA cycle. The fold-changes between *Oxr1*^*+/+*^ and *Oxr1*^*d/d*^ mice of the indicated metabolites are colour-coded. The boxes indicating putative metabolites are surrounded in red. The boxes indicating non-identified metabolites are show in white. *p < 0.05, **p < 0.01, and ***p < 0.001 shown in each box represent the significance of the changes for a given metabolite. (PNG 100 kb)

High-resolution image (TIF 314 kb)

Supplementary Fig. 3 Expression levels of *Gpi1* (top panel), *Oxr1-FL* (middle panel), or *Oxr1-C* (bottom panel) in N2a cells transfected with the indicated vectors as determined by qRT-PCR. (*p < 0.05, **p < 0.01, and ***p < 0.001, one-way ANOVA as compared to corresponding controls (grey bars) either shRNA scramble, empty vector, or shRNA scramble plus empty vector). (PNG 36 kb)

High-resolution image (TIF 181 kb)
