# Supplementary figures and images for: Oxidation Resistance 1 Modulates Glycolytic Pathways in the Cerebellum via an Interaction with Glucose-6-Phosphate Isomerase
Source: Mol Neurobiol. 2018 Jun 15;56(3):1558–77. doi: 10.1007/s12035-018-1174-x (PMC6368252; doi:10.1007/s12035-018-1174-x)

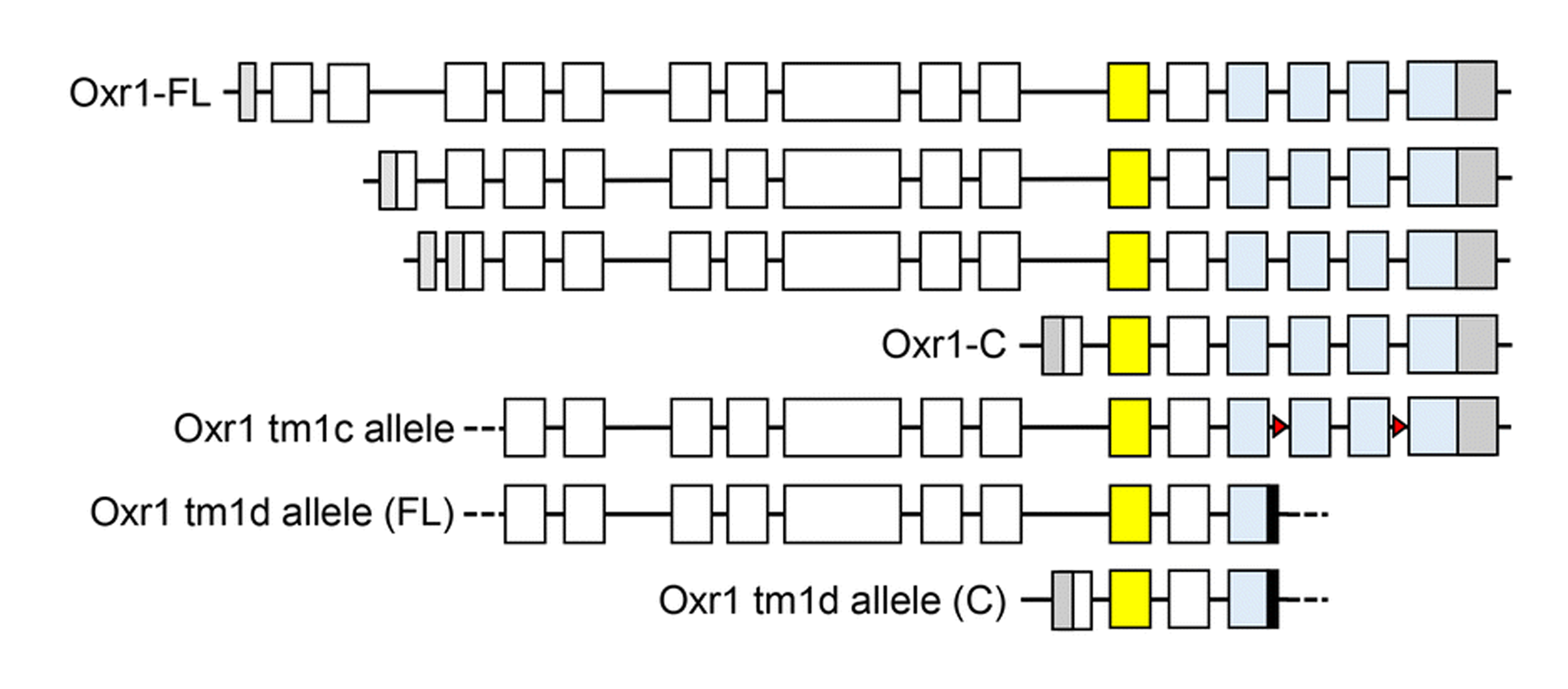

Supplement: Supplementary file 3 — Schematic of mouse Oxr1 isoforms based on the UCSC Genome Browser (GRCm38/mm10) showing the range of annotated full-length (FL) isoforms with alternative start sites and the shortest isoform (c). Coding exons (white), UTRs (grey), an alternatively-spliced exon (yellow), and the TLDc domain (blue) are shown. Not to scale. The Oxr1 knockout allele (tm1d) used in this study is also indicated that truncates all of the above isoforms. This allele was generated by from the corresponding tmlc allele with loxp sites (red arrows) flanking two coding exons in the TLDc domain. (PNG 41 kb) [file 12035_2018_1174_Fig8_ESM.png]

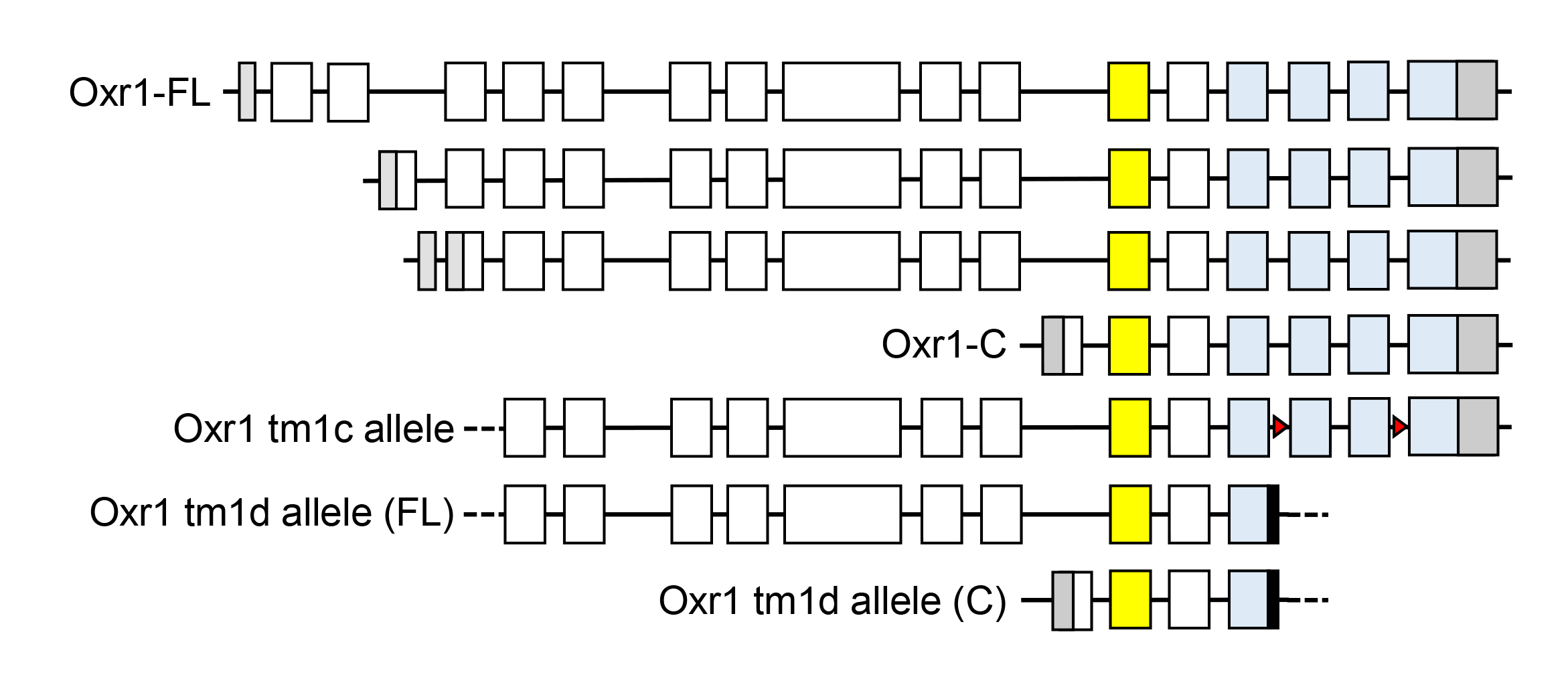

Supplement: Supplementary file 4 — High-resolution image (TIF 136 kb) [file 12035_2018_1174_MOESM3_ESM.tif]

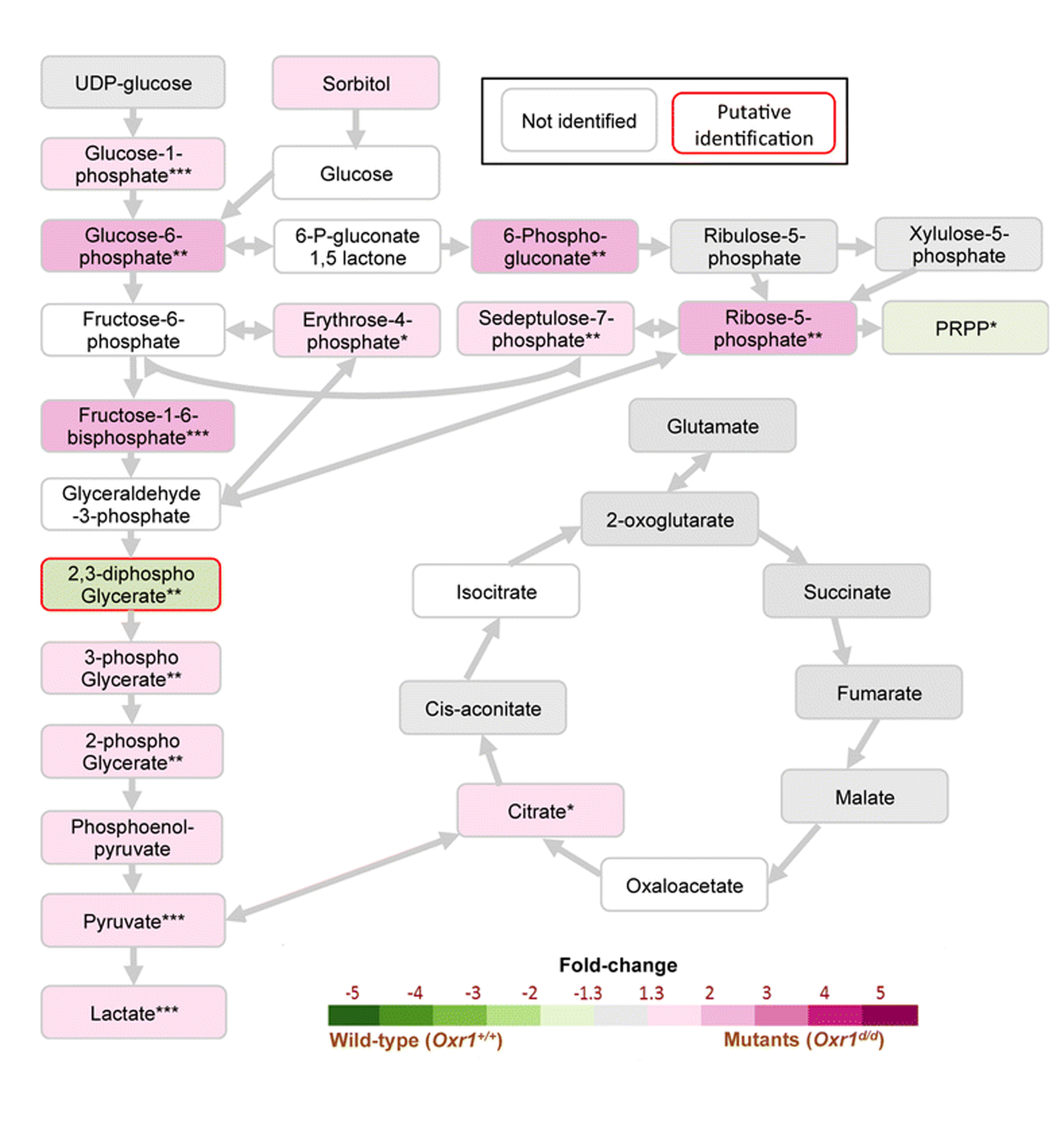

Supplement: Supplementary file 5 — Pathway analysis of the metabolites dysregulated in the Oxr1d/d cerebellum. These metabolites were mainly from the glycolysis, pentose phosphate pathway, and the TCA cycle. The fold-changes between Oxr1+/+ and Oxr1d/d mice of the indicated metabolites are colour-coded. The boxes indicating putative metabolites are surrounded in red. The boxes indicating non-identified metabolites are show in white. *p < 0.05, **p < 0.01, and ***p < 0.001 shown in each box represent the significance of the changes for a given metabolite. (PNG 100 kb) [file 12035_2018_1174_Fig9_ESM.png]

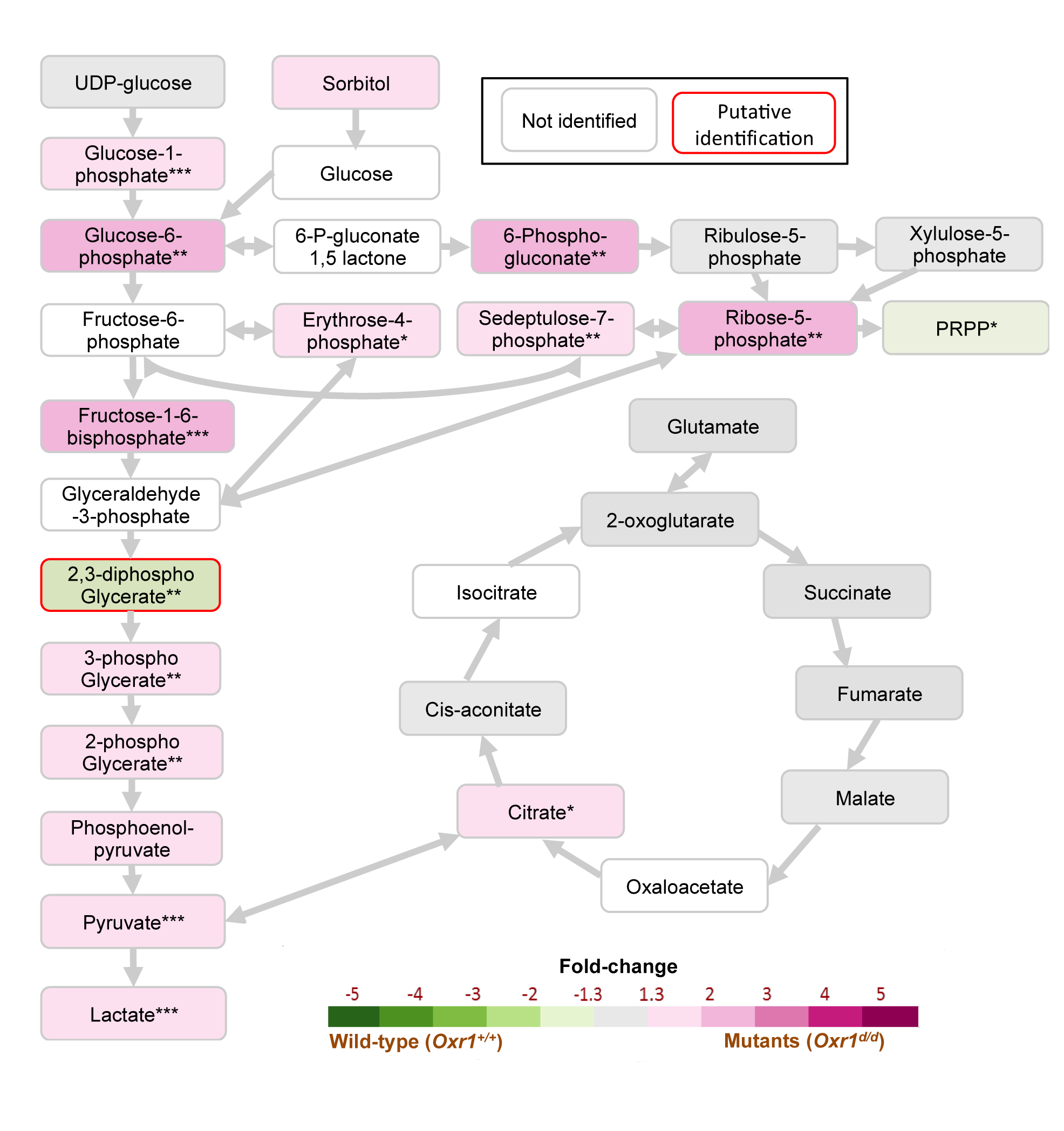

Supplement: Supplementary file 6 — High-resolution image (TIF 314 kb) [file 12035_2018_1174_MOESM4_ESM.tif]

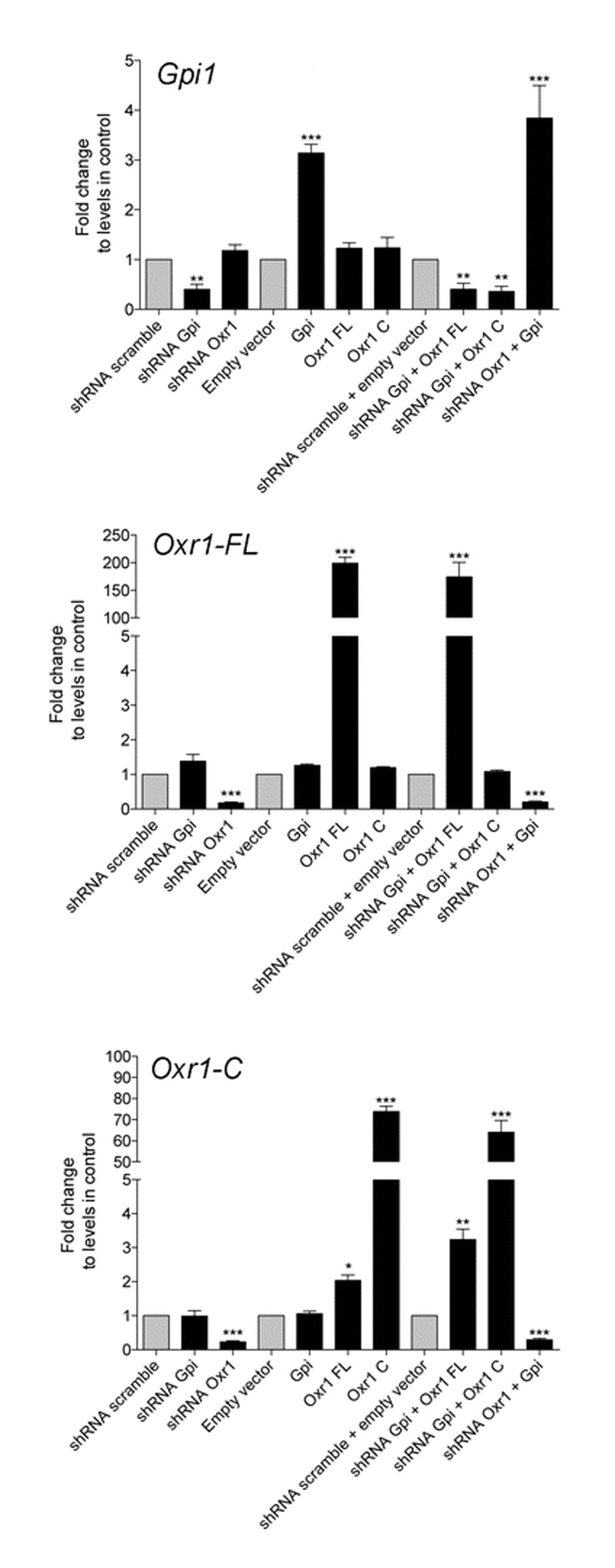

Supplement: Supplementary file 7 — Expression levels of Gpi1 (top panel), Oxr1-FL (middle panel), or Oxr1-C (bottom panel) in N2a cells transfected with the indicated vectors as determined by qRT-PCR. (*p < 0.05, **p < 0.01, and ***p < 0.001, one-way ANOVA as compared to corresponding controls (grey bars) either shRNA scramble, empty vector, or shRNA scramble plus empty vector). (PNG 36 kb) [file 12035_2018_1174_Fig10_ESM.png]

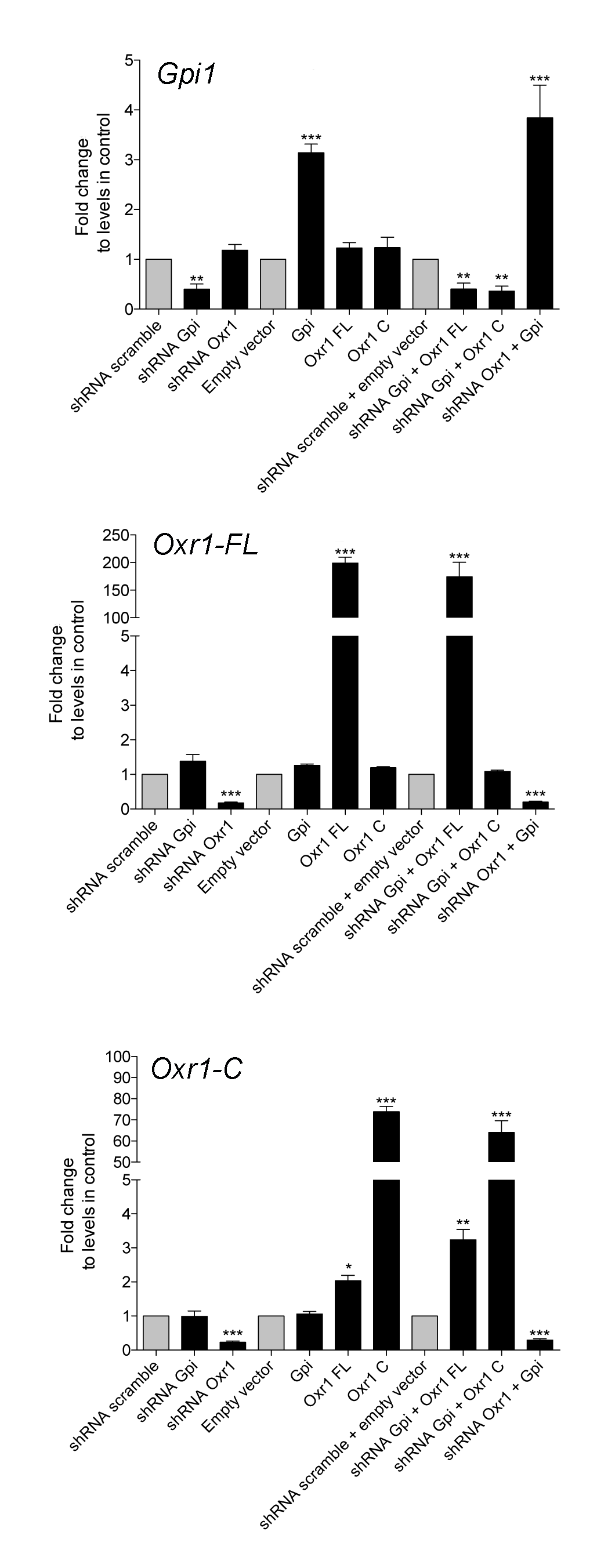

Supplement: Supplementary file 8 — High-resolution image (TIF 181 kb) [file 12035_2018_1174_MOESM5_ESM.tif]
